# The inner elbow skin microbiome contains *Lactobacillus* among its core taxa and varies with age, season and lifestyle

**DOI:** 10.20517/mrr.2024.23

**Published:** 2024-08-29

**Authors:** Lize Delanghe, Ilke De Boeck, Joke Van Malderen, Thies Gehrmann, Camille Nina Allonsius, Peter A. Bron, Ingmar Claes, Margo Hagendorens, Julie Leysen, Stijn Wittouck, Sarah Lebeer

**Affiliations:** ^1^Department of Bioscience Engineering, University of Antwerp, Antwerpen 2020, Belgium.; ^2^YUN NV, Niel 2845, Belgium.; ^3^Department of Pediatrics, University Hospital Antwerp/University of Antwerp, Edegem 2650, Belgium.; ^4^Department of Dermatology, University Hospital Antwerp/University of Antwerp, Edegem 2650, Belgium.

**Keywords:** Skin microbiome, metagenomic shotgun sequencing, lactobacillus

## Abstract

**Background:** The human skin microbiome plays an essential role in protecting against pathogens and other external substances. This open ecosystem is also influenced by personal and environmental factors, but the precise impact of these factors, such as lifestyle and season, is understudied. We focused here on the inner elbow, a skin site prone to inflammatory conditions like atopic dermatitis and psoriasis.

**Methods:** We collected skin swabs from the inner elbow of 52 children and adults, with no signs of skin disorders, in the winter and summer seasons. Samples were analyzed using metagenomic shallow shotgun sequencing. In addition, metadata were collected using questionnaires on health, lifestyle, and environmental factors.

**Results:** The core inner elbow community, taxa with a prevalence of 95% or higher, consisted of several well-known skin taxa, such as *Staphylococcus hominis*, *Staphylococcus capitis*, *Staphylococcus epidermidis*, and *Cutibacterium acnes*. In addition, *Streptococcus* and *Lactobacillus* species were also found to be highly prevalent members of the skin microbiota, especially in the age group up to 3 years old. Of all investigated factors, age appeared to be the major driver defining the skin microbiome composition and longitudinal stability over the seasons. Differential abundance analysis using three statistical tests also pointed out that specific skin species were significantly associated with sampling season, age, hygiene practices, vitamin D supplements, probiotics, and the number of household members.

**Conclusion:** This study identifies novel factors influencing the inner elbow skin microbiome composition and paves the way for future comparative and intervention studies in skin disorders such as atopic dermatitis.

## INTRODUCTION

The skin is one of the largest organs of the human body in terms of surface area, and the first interface between the external environment and internal body. Together with its microbiota, it has an essential role in protecting against invading pathogens and other foreign substances^[1]^. Commensal skin bacteria can exert this protective role through different mechanisms such as skin-barrier strengthening^[2]^, pathogen exclusion, and education and regulation of the local and systemic immune system^[3,4]^. Several tools are available to study the skin microbiota composition *in situ*, with metagenomic shotgun sequencing being preferred over 16S rRNA gene amplicon sequencing, as it allows a more detailed taxonomic identification up to species or strain level and a more accurate functional annotation. However, metagenomic shotgun sequencing is costly and has some technical challenges, especially related to bacterial DNA extraction from low-biomass skin samples. It is important to be aware of these challenges and the associated bias^[5]^. Up to now, 16S amplicon sequencing remains the most popular technique for skin microbiome analysis, mostly because of the low costs and reproducibility for low-biomass samples. However, because of its advantages in taxonomic and functional resolution, metagenomic shotgun sequencing has become increasingly popular over the last few years^[6]^.

The skin harbors different physiological sites, resulting in different bacterial communities^[7]^. For example, a 16S rRNA sequencing study in 110 adult men revealed that *Cutibacterium*, *Staphylococcus*, and *Corynebacterium* are more abundant in the axilla and scalp than on the forearm*,* while the inner arm typically harbors a more diverse microbiome^[8]^*.* On oily skin sites (e.g., face), Cutibacteria such as *Cutibacterium acnes* (*C. acnes*) are mainly found^[9]^*.* Based on descriptive microbiome studies*,* personal (e.g., host genetics^[10]^) and environmental factors (i.e., age, ethnicity, environmental temperature and moisture, hygiene practices, cosmetics, and diet) have also been suggested to impact the skin microbiome^[11]^. The influence of age on both alpha and beta diversity levels is well established. For example, a study in 31 healthy infants (age 3-52 weeks) based on pyrosequencing of the volar forearm, buttock, and forehead showed that richness and evenness increase with age^[12]^. The same authors observed a dominance of *Staphylococcus* and *Streptococcus* in the youngest infants, which decreases as the infant ages. Similar findings were also found in a more recent study (*n* = 48) using whole-genome shotgun sequencing, where a shift in facial skin microbial diversity and increase in *C. acnes* was observed in early and late stages of puberty, coinciding with increased facial sebum production^[13]^. Although several studies investigated the healthy skin microbiome at sites that are highly prone to diseases, such as the inner elbow for atopic dermatitis^[14]^ and psoriasis^[15]^, they mostly focused on different age groups and sampling time points, and we currently lack in-depth data on the role of other lifestyle and environmental factors^[16-18]^. More detailed information on the skin microbiome and specific associations up to the species level is necessary in order to design human intervention studies with microbiome-targeting therapies. For example, we and others have previously found indications for positive associations between lactobacilli and skin health, such as when applied exogenously on acne patients^[19]^, but their native presence on different body sites is currently not well studied. Therefore, in this study, we will further investigate the presence of lactobacilli in the healthy inner elbow skin microbiome. In addition to topical microbiome-acting therapies, there is also an increased interest in modulating the skin microbiome with oral strategies such as oral food supplements with vitamin D^[20]^ and probiotics^[21]^, but their association with the skin microbiome is currently understudied in cohort studies.

In this study, we studied the intact skin microbiome (with no eczema or other skin conditions at the time of sampling) of the inner elbow of a cohort in Belgium (*n* = 52) by metagenomic shallow shotgun sequencing during the winter and summer seasons. We also collected relevant metadata via an integrated questionnaire to study the association between lifestyle and environmental factors and skin microbiome.

## METHODS

### Human subjects

Human subjects (aged above 6 months) were recruited via email and personal contacts, and children were recruited through contact with their parents or legal guardians. In total, 52 healthy volunteers (63.5% female, average age 12) participated in the study. Four participants had been excluded because of previous atopic dermatitis symptoms. The total age range was 0 to 53 years, with the following numbers for each age group: 11 of 0-3 years, 10 of 3-6 years, 11 of 6-12 years, 10 of 12-18 years and 10 of more than 18 years old. The only exclusion criteria were self-reported presence of atopic dermatitis symptoms or other skin conditions, use of topical corticosteroids and/or antibiotics within two weeks before sampling, and use of oral antibiotics within four weeks before sampling. This study was approved by the Ethics Committee of the Antwerp University Hospital/University of Antwerp (registration number B3002020000099, approved 25 May 2020, ClinicalTrials.gov Identifier: NCT04771910). Written informed consent was obtained from all participants (or in the case of minors, their parents/legal guardians) prior to sampling. Supplementary Table 1 gives an overview of participants’ demographics.

### Sample collection

The first skin swabs were collected between 15 and 31 March 2021 and the second swabs between 30 May and 22 June 2021. All participants received a detailed instruction booklet on how to self-sample skin samples in a standardized way. For children under 16, a parent or other caretaker helped with the sampling. Skin samples in the elbow bend were collected with the eNAT swab (Copan, Brescia, Italy). Participants were required to not wash the elbow region for 8 h before sampling, as well as not to use creams or other cosmetics on the elbow. Skin samples were collected by turning around the swab while brushing the area of the elbow bend for 30 seconds. Prior to sampling, the eNAT swab was soaked in sterile pre-moisture buffer (50mM Tris buffer [pH 7.6], and 0.5% Tween-20). The swabs were transferred immediately after sampling to the vial that contained the eNAT buffer and stored at 4 °C for a maximum of two weeks and at -20 °C for a maximum of 6 months until further processing. In total, we collected 104 skin swabs from our 52 participants.

### Metadata collection

At the first sampling time point, all participants (or their parents/legal guardians) were asked to fill out a questionnaire on multiple general health, lifestyle, and environmental factors through the online survey system Qualtrics [Supplementary Questionnaires]. At the second sampling time point, 12 weeks later, participants were asked to fill out a short questionnaire to investigate which factors changed during the past 12 weeks [Supplementary Questionnaires].

### Microbial DNA extraction

Skin swabs were stored at -20 °C for periods ranging from 2 weeks to almost 6 months. DNA was extracted with the DNeasy PowerSoil Pro kit (Qiagen, Hilden, Germany) according to the manufacturer’s instructions, involving both chemical (lysis buffer, provided in the kit) and mechanical lysis. The latter was performed via bead beating on a benchtop vortex with bead adaptor. Prior to the DNA extraction, all swabs were vortexed for 15-30 s. DNA concentration of all samples was measured using the Qubit 3.0 Fluorometer (Life Technologies, Ledeberg, Belgium) according to the manufacturer’s instructions.

### Metagenomic shotgun sequencing

Library preparation for metagenomic shotgun sequencing was performed using the Illumina DNA Prep (Illumina, San Diego, California, United States) according to the manufacturer’s instructions and as described before^[5]^. DNA concentrations, pooled volumes, and library sizes of all samples are shown in Supplementary Table 2. After library preparation, 5 µL of the library was denatured with 0.2N NaOH (Illumina), diluted to 10 pM and spiked with 10% PhiX control DNA (Illumina). Dual-index paired-end sequencing was performed using a MiSeq Reagent Kit V2 (300 cycles) (Illumina) and MiSeq Desktop sequencer (M00984, Illumina).

### Bioinformatic analysis

Metagenomic shotgun reads were filtered with the dada2 R package. Reads shorter than 50 base pairs or reads with more than two expected errors were removed. Next, read pairs were classified with Kraken2 using the MiniKraken2 v2 reference database. This database was constructed from the bacterial, archaeal, and viral genomes in NCBI RefSeq, in addition to the human genome version GRCh38 (to detect human contamination). Reads classified as non-bacterial taxa were removed. Based on these classifications, a read count table was constructed where the columns represent taxa and the rows represent samples. Taxa were either species or higher-level taxa for reads that were unclassified at one or more ranks. Samples contained, on average, 13,517 high-quality bacterial reads per sample. Parsing of Kraken2 output was implemented in R version 4.2.1.

Since there was a focus on a core microbiome analysis in our work and such an analysis is sensitive to even small amounts of contamination, we assessed the presence of genera with a prevalence of at least 95% (candidate core genera) as well as *Lactobacillaceae* species with a prevalence of at least 20% in our four DNA extraction negative controls. Based on this inspection, the genera *Bradyrhizobium*, *Methylobacterium*, and *Sphingomonas*, as well as the species *Lacticaseibacillus rhamnosus*, were removed from the data.

The presence of clusters in our sample cohort was tested by using the prediction metric^[22]^. Prediction strength was evaluated to confirm the presence or absence of specific clusters in our sample cohort for both hierarchical (left) and PAM clustering (right). Prediction strength was measured based on four distance matrices: Bray-Curtis (on relative abundances), Bray-Curtis on the presence/absence level, Jensen-Shannon divergence, and Jense-Shannon distance (equal to the square root of the Jensen-Shannon divergence). For hierarchical clustering, moderate support was only observed for one distance matrix. For PAM clustering, no support was observed for significant clusters in our dataset.

Differential abundance analysis was performed using the in-house R package multidiffabundance, version 0.0.1 (publicly available at https://github.com/thiesgehrmann/multidiffabundance). It implements seven differential abundance testing methods and reports the consensus, which is more reliable than the result of any individual testing method (https://www.nature.com/articles/s41467-022-28034-z). In this paper, as we tested random effects, only three methods could be used: Limma^[23]^, Maaslin2^[24]^, and linear regression on the centered log-ratio transformed taxa abundance. Spurious taxa were filtered out based on presence in at least one sample with 0.25% relative abundance. Using the same package, we tested the effects upon alpha diversity (Shannon diversity) with a linear regression and beta diversity (using the adonis2 function implemented in the vegan package)^[25]^. In each method, we tested the formula: ~ effect + pooled_volume + time point + antibiotics + sex + (1|participant), where the effect is the tested effect of interest, and antibiotics refer to antibiotic use in the last three months. As diversity measures, alpha and beta diversity were visualized. For alpha diversity or the diversity of taxa within the different skin samples, two measures were used: (1) observed diversity or the number of taxa, and (2) the Inverse Simpson index, which also accounts for the evenness of the community. To measure beta diversity or the diversity between the different skin samples taken here, the species composition between the two samples was compared by calculating Bray-Curtis dissimilarity and visualizing the samples in a PCoA plot.

To identify taxa that belong to the core elbow skin microbiome, we set a threshold of presence in 95% of samples. In addition, to check the possible effect of barcode leakage and cross-contamination on the prevalence of taxa, we only defined genera to be part of the core elbow skin microbiome if they had a higher concentration (library size/pooled volume) in all samples compared to the controls.

All samples were sequenced on two sequencing runs, with the samples of both time points from one participant grouped on the same run to exclude technical variation as a reason for detecting intrapersonal differences. Negative controls for DNA extraction and library preparation were taken. The microbial composition of the negative controls can be found in Supplementary Figure 1. Quality control was performed by filtering samples with read counts equal to or lower than the negative controls. 92.3% of the samples passed the quality control pipeline and we obtained high-quality profiles of 48 participants. For AHD010, AHD011, ADH034, and ADH037, the samples of time point 0 were excluded because of too low read counts.

## RESULTS

### Presence of a core microbiome on the elbow skin that includes *Lactobacillus* taxa

Samples from 52 healthy participants were sequenced by shallow shotgun sequencing at an average of 111 567 (SD 111 078) total reads per sample, and 13 945 (SD 13 885) bacterial reads per sample to allow identification up to species level for most taxa. Supplementary Table 1 gives an overview of participants’ demographics and total and bacterial read counts for each sample are shown in Supplementary Table 3. A thorough inspection of four negative control samples [Supplementary Figure 1] resulted in the removal of four taxa from the data [Supplementary Figures 2 and 3].

**Figure 1:**
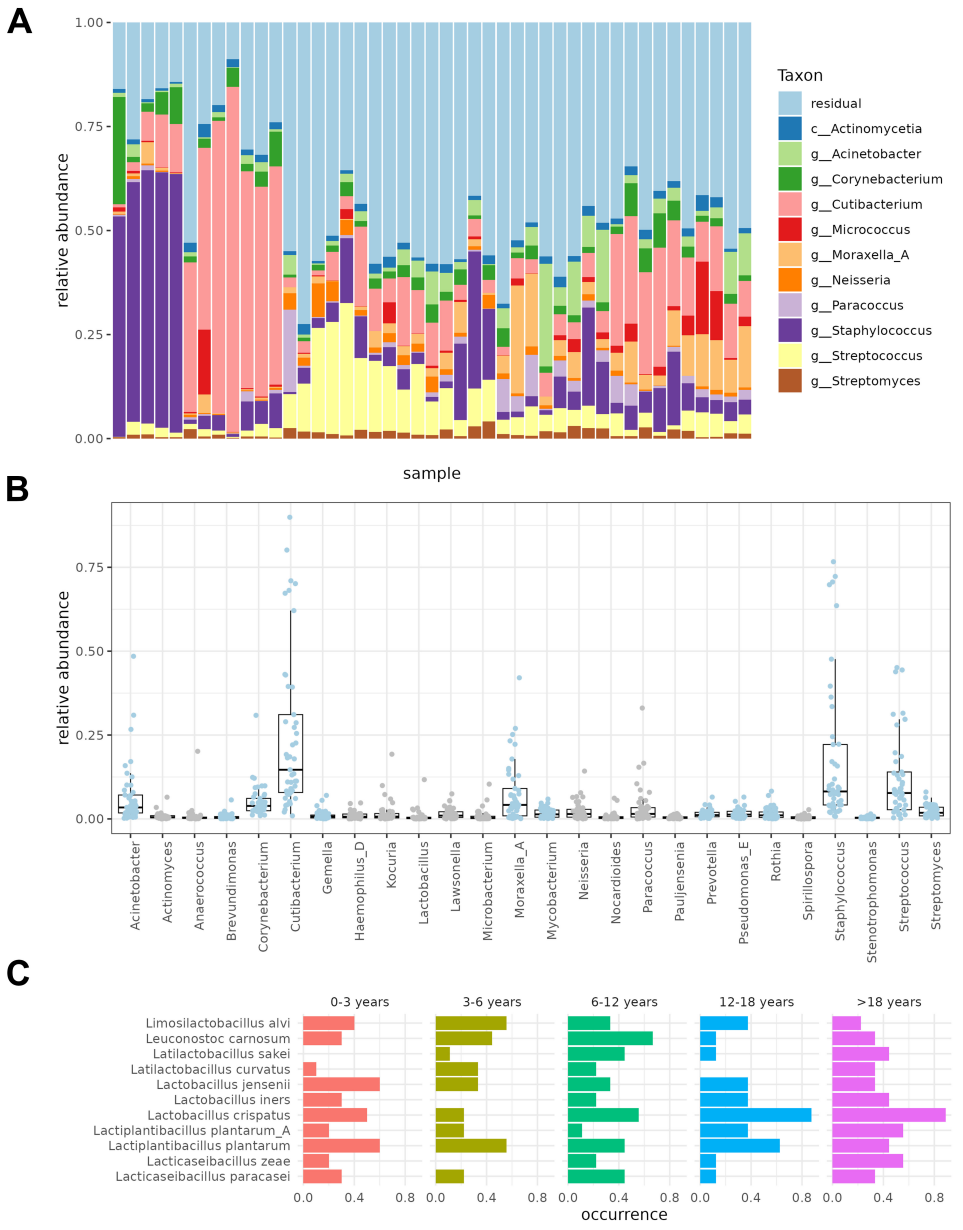
The microbial composition of healthy elbow skin (A) Bar chart describing the microbial composition of all skin samples at time point 0, at the genus level and ordered by hierarchical clustering based on Bray-Curtis similarities; (B) Boxplots of the relative abundances at baseline (T0) of the core genera with a prevalence of 95% or higher. Blue indicates a prevalence of 100%; (C) Bar chart showing the occurrence at baseline (T0) of the Lactobacillaceae species with an occurrence of at least 20% in all samples.

The skin microbiome communities of all collected skin samples were mainly represented by the taxa *Acinetobacter* (2.1%), *Corynebacterium* (2.1%), *Cutibacterium* (10.0%), *Micrococcus* (0.5%), *Moraxella_A* (2.3%), *Neisseria* (0.8%), *Paracoccus* (1.0%), *Staphylococcus* (4.6%), *Streptococcus* (4.2%), and *Streptomyces* (1.1%) [Figure 1A]. The class with the highest relative abundance was *Actinomycetia* (31.0%). Hierarchical clustering of the microbial profiles at the species level revealed no discrete clusters in our sampled cohort [Supplementary Figure 4], suggesting the presence of a shared core skin microbiome in our cohort.

Next, the core elbow skin microbiome was evaluated at baseline (T0), which we defined as all taxa having a prevalence of 95% or higher. We found 14 genera with a prevalence of 100% in our population, so present in all samples [Figure 1B]. Of these 14 genera, *Acinetobacter* (3.70%), *Cutibacterium* (16.7%), *Moraxella* (4.31%), *Staphylococcus* (12.5%), *Corynebacterium* (3.43%), and *Streptococcus* (6.96%) showed the highest relative abundance. Of note, these 14 genera with a 100% prevalence also included taxa that were consistently present, but at rather low relative abundance, such as *Brevundimonas* (0.368%), Mycobac*terium* (1.03%), *Prevotella* (0.857%), and *Rothia* (0.935%) [Figure 1B]. When looking at the species level, *C. acnes* (14.4%), *Staphylococcus hominis* (6.25%), *Staphylococcus capitis* (2.36%), *Staphylococcus epidermidis* (1.34%), and *Micrococcus luteus* (1.41%) had the highest relative abundances with prevalences between 89% and 100% [Supplementary Table 4].

In addition to these 14 omnipresent genera, we also detected 12 genera with a high prevalence of 95% or higher, thus also part of the core elbow skin microbiome [Figure 1B]. These core taxa included the *Lactobacillus* genus, with *Lactobacillus crispatus*(*L. crispatus*), *Lactobacillus iners* (*L. iners*), and *Lactobacillus jensenii* (*L. jensenii*) identified as the most prevalent species within this genus, with prevalences of 60.0%, 26.7% and 40.0%, respectively [Figure 1C]. *Lactobacillus* species displayed the highest relative abundance in the age groups 0-3 years old (median 0.232%), 12-18 years (median 0.250%), and > 18 years old (median 0.299%) [Supplementary Figure 5]. In addition, we also identified nomadic lactobacilli *s*pecies, such as *Lactiplantibacillus plantarum*, to be present in 53.3% of samples with an average relative abundance of 0.212%. Two other species of *Lactobacillaceae*, *Leuconostoc carnosum* and *Limosilactobacillus alvi*, were found on the skin in this dataset*,* with notably both a prevalence of 37.8% and relative abundances of 0.0602% and 0.0281%, respectively [Figure 1C].

### Age and season are major factors determining the skin microbiome composition

**Figure 2:**
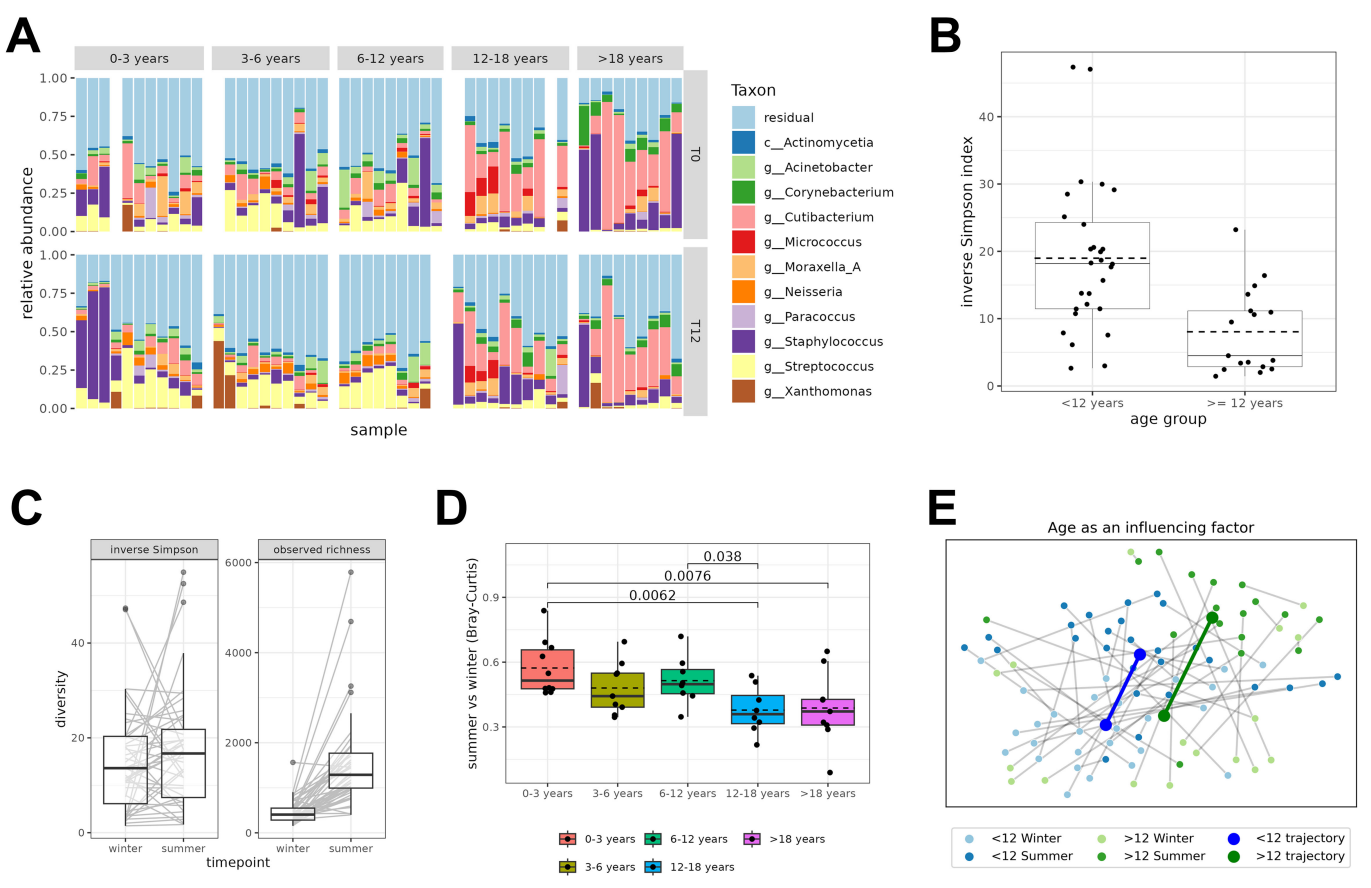
Skin microbiome composition between different age groups. (A) Individual bar charts for taxonomic bacterial community composition per participant for the winter and summer time points. White spaces are samples that dropped out after quality control; (B) Inverse Simpson Index for skin swabs of healthy subjects below 12 years old and from 12 years and older (at baseline, T0) at the genus level. Dashed lines represent the mean Inverse Simpson index; (C) Inverse Simpson index and observed richness for skin samples during the winter and summer seasons at the genus level; (D) Bray-Curtis dissimilarities between each taxonomic profile and the profile from the same participant during the winter vs. summer seasons for different age groups. Only significant p-values are visualized in the figure (Wilcoxon test); (E) PCoA plot distributing the samples according to beta diversity for both seasons and age groups. Samples are colored by age group (< 12 and > 12 years old) and season (winter and summer). The average trajectory for each age group is visualized by the thick lines. Statistics were performed with the Wilcoxon test.

Since we observed an association with age for the relative abundance of *Lactobacillus* species [Figure 1C], we aimed to further zoom in on age as a possible key factor shaping the skin microbiome composition. In our cohort, at least 10 participants were included in each of five predefined age groups (0-3 years, 3-6 years, 6-12 years, 12-18 years, and over 18 years old). When comparing the overall skin microbiome composition in all age groups, we observed a clear shift at the age of 12 years, mainly characterized by a higher relative abundance of *Cutibacterium* [Figure 2A]. Therefore, we analyzed the alpha diversity for the participants below and above 12 years old. The inverse Simpson index was significantly lower in the elbow skin samples of participants of 12 years and older compared to the younger group (Figure 2B, *P* = 1.6 × 10^-4^), while no differences were observed for genus richness [Supplementary Figure 6]. This suggests that this older group has a rather uneven distribution of taxa, with a limited number of dominant bacterial taxa. In contrast, the elbow microbiome of children under 12 years old seemed to be characterized by a more evenly distributed, broader palette of taxa.

To evaluate the impact of season as an important another possible driving factor, the samples collected during the winter season were compared with paired samples from the same participant collected in the summer season. Alpha and beta diversity measures were calculated to estimate the differences in bacterial diversity between these different time points. The observed richness significantly changed between the two time points (Figure 2C, *P*-value = 2.2 × 10^-13^), with an average of 458 observed genera at the start (winter season) and 1,537 genera at T12 (the summer season). The Inverse Simpson index was not significantly different (with average values of 14 and 17 in the winter and summer seasons, respectively) [Figure 2C].

**Figure 3:**
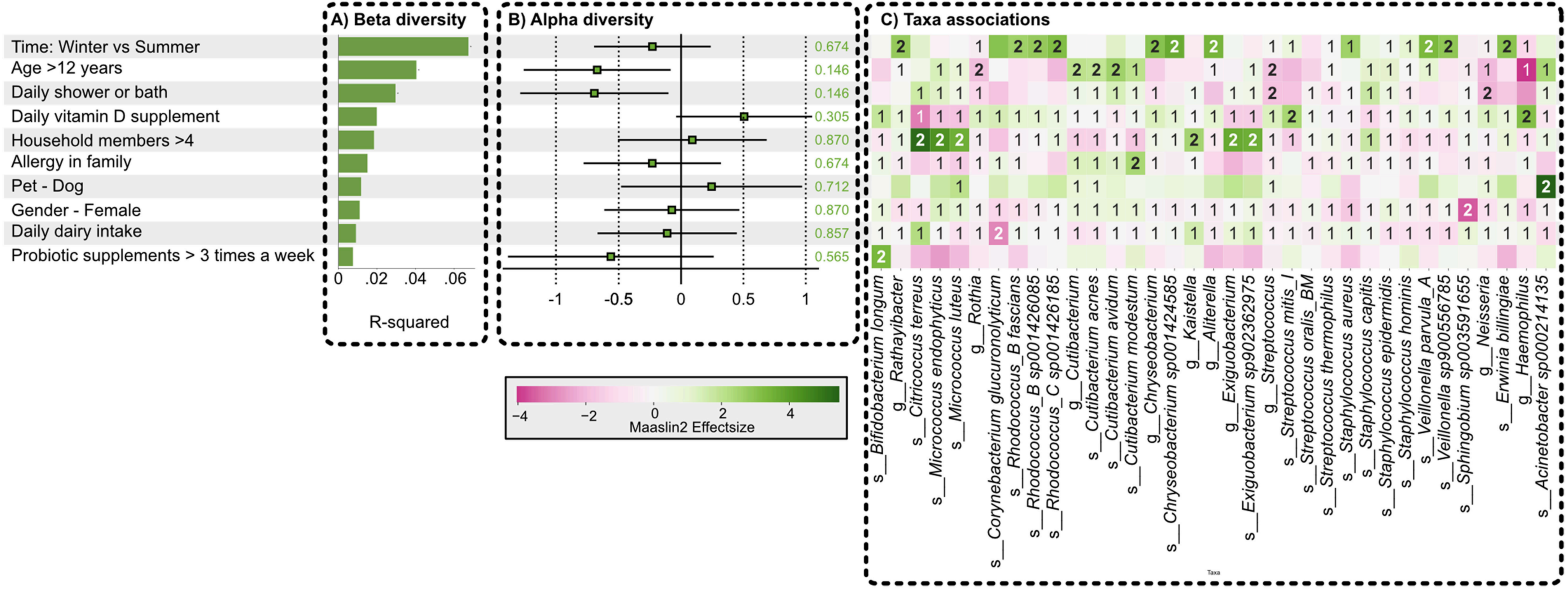
Statistical analysis of the association of different personal, lifestyle, health, hygiene, and environmental factors with the skin microbiome. Each panel displays associations on different levels of the microbiome: (A) associations on the level of beta diversity between the samples, significant associations indicated with *(*P*-value < 0.05, Adonis test); (B) associations on the level of alpha diversity of the samples; (C) associations on the level of abundances of specific taxa as analyzed by 3 different differential abundance testing methods (Limma, Maaslin2, and a regression on the centered log-ratio transformed abundance data). All taxa are shown to have a significant association for at least one statistical test. Numbers indicate the number of tests that measured significant differential abundance.

We also observed higher stability over the seasons of the skin microbiome profiles of participants of 12 years and older [Figure 2A], which could be related to the higher dominance of *C. acnes*. Therefore, we calculated beta diversity by Bray-Curtis (BC) dissimilarity between the winter and summer time points for the five different age groups. The BC dissimilarity was indeed significantly lower in the age groups 12-18 years old and older than 18 years compared to the three younger age groups [Figure 2D]. The beta diversity was also visualized in a Principal Coordinates Analysis (PCoA) plot [Figure 2E]. This figure showed the average movement over time of all individual participants at the two time points. Samples of winter and summer were clearly represented by two distinct clusters (light and dark colors). In addition, distinct clusters were also observed based on age (blue: < 12 years and green: > 12 years old) [Figure 2E]. When exploring the effects of age and season on the bacterial community with Adonis, beta diversity was significantly associated with both age (R^2^ value 0.0371) and season (R^2^ value 0.0572), with a higher R^2^ value for season (Figure 3, first column).

In addition to the diversity analysis, we investigated whether specific taxa showed a differential abundance and performed these analyses for taxa that were present in at least 30% of all samples using three differential abundance tests (Limma, Maaslin2, and a linear regression on the centered log-ratio transformed taxa abundance) and visualized the results for all taxa having at least one significant association (Figure 3, third column). First, we looked into the differential abundance of specific taxa between the age groups (below and above 12 years old) and seasons. A significant association was especially found between an age above 12 and a higher relative abundance of *C. acnes*, *Cutibacterium avidum* and a lower abundance of *Streptococcus mitis* (*S. mitis*), *Neisseria* (not further classified), and *Haemophilus* (not further classified). Next, we looked at seasonal impact. The taxa that were more prevalent in samples collected during the summer season compared to those collected in winter showed a higher abundance, including *Rhodococcus* and *Veillonella* taxa, based on at least 2 of 3 statistical tests.

### Lifestyle factors associated with individual bacterial taxa of the skin

We then aimed to quantify associations between different parameters of the skin microbiome profiles and the lifestyle information we collected via questionnaires from the subjects on several host-related and lifestyle variables [Figure 3]. This questionnaire recorded hygiene practices, family history of eczema, diet, pets, and antibiotic intake as known factors that impact the skin microbiome^[8,26,27]^. In addition, we included survey questions on other variables for which we postulated a possible association, but for which limited data were available in the literature: use of oral probiotics, intake of fermented foods, number of household members, and Vitamin D intake [Supplementary Questionnaires].

We found that daily shower/bath (R² value 0.0244) or not was shown to have a significant association with the elbow skin microbiome profiles at the beta diversity level, as evaluated with an Adonis test (Figure 3, first column). Of interest, these factors did not significantly impact the alpha diversity (Shannon diversity) of our samples, as analyzed with a multiple linear regression where also the potential confounders sample volume, time point, antibiotic use, and sex were included (Figure 3, second column). This indicates that these factors influence the relative abundance of specific taxa or groups of taxa, altering the overall composition of the microbial community, while keeping the total number of taxa and evenness relatively constant. Therefore, we also analyzed the effect of several lifestyles on the differential abundance of specific taxa. Daily vitamin D supplementation was associated with a significantly higher abundance of *Bifidobacterium longum* (1 statistical test)*,* and *S. mitis* in the skin samples. Living together with more than 4 household members was associated with a significant increase in taxa such as *Citricoccus terreus*, *Micrococcus luteus* and *Micrococcus endophyticus*. Taking a shower or bathing daily was associated with a reduced relative abundance of *Streptococcus* taxa and an increased relative abundance of *Staphylococcus capitis* and *Staphylococcus epidermidis* compared to participants who did not have this daily routine*.* Of interest, probiotic supplements appeared to increase the relative abundance of *Bifidobacterium longum* on the skin.

## DISCUSSION

In this study, we characterized the skin microbiome of the inner elbow of a Belgian population with no signs of eczema or other skin disorders (self-reported) by metagenomic shotgun sequencing. Factors that could shape the skin microbiome composition such as the season, age and different health, lifestyle and environmental factors were associated with the different parameters of the elbow skin microbiome composition.

Fourteen genera were identified, all of which showed a prevalence of 100% in our population. Among these, *Staphylococcus*, *Cutibacterium*, *Acinetobacter*, *Streptococcus*, and *Corynebacterium* are well documented as skin genera^[7]^. *Moraxella*, *Brevundimonas*, *Rothia*, *Prevotella*, *Stenotrophomonas*, *Streptomyces*, *Pseudomonas*, *Gemella*, and *Mycobacterium* have not previously been widely described as core skin taxa. They were sometimes found in specific skin microbiome studies, e.g., in studies about the healthy and atopic dermatitis skin microbiome^[12,28,29]^, but are not yet generally considered as important skin microbiome members. These differences between our Belgian cohort and other published skin microbiomes, in, for example, the USA^[8,9]^, could be due to the differences in these geographical areas^[30]^, which might result in distinctly different environmental holobiomes feeding into the skin microbiome. Of course, it can also be due to technical differences, such as sampling methods and storage and sequencing protocols and techniques^[5]^. By including negative controls, checking the possible effects of barcode leakage and cross-contamination, performing library preparation with the same kit for all samples, and carefully dividing samples over the two sequencing runs to not split up samples from the same participant, we tried to exclude as much as possible technical and environmental influences.

We identified different low-abundant genera with a 100% prevalence, such as *Brevundimonas*, *Rothia*, and *Prevotella*. These taxa could be further investigated as potential keystone species, defined as taxa exerting a considerable influence on microbiome structure and functioning irrespective of their (lower) abundance across space and time^[31]^. Of interest is *Lactobacillus*, which was shown to have a prevalence of 95% on the elbow. In previous research, we also found that *Lactobacillaceae* represent important members of the cheek microbiome^[32]^. The facial/cheek skin is close to the mouth and *Lactobacillaceae* are frequently present in fermented foods, so their presence in areas near the mouth could also result from temporary colonization from food origin. The fact that we now also observed lactobacilli on the inner elbow skin, in addition to other studies that also found lactobacilli on sebaceous, moist, and dry skin sites^[8,9]^, corroborates the notion of lactobacilli as common inhabitants of skin sites throughout the human body. Inspection of the specific taxa detected on the elbows in our study cohort revealed species such as *L. crispatus*, *L. iners*, and *L. jensenii* as the most dominant lactobacilli taxa, with a significantly higher relative abundance in the youngest age group (6 months up to 3 years old) compared to the age group of 3 to 6 years old. This is consistent with the fact these taxa are among the first colonizers of a newborn upon a vaginal delivery^[33,34]^, since these taxa dominate in the vagina^[35]^ and their relative abundance is further increased during pregnancy^[36]^. At 12 years old, the relative abundance of the *Lactobacillus* genus increases again, which could, for example, be explained by the changing physiology of the skin at puberty, changing hormonal levels, and increased sexual contacts, but this remains to be further studied. In addition, we also found more nomadic taxa such as *Lactiplantibacillus plantarum* and *Leuconostoc carnosum* to be consistently present in our data. Notably, a *Lactiplantibacillus plantarum* strain was recently formulated in a skin cream with *Lacticaseibacillus rhamnosus* and *Lactiplantibacillus pentosus* and first shown to reduce skin pathobionts activity, inflammation, and acne symptoms in an open-label, and then validated in a larger double-blind placebo-controlled trial^[32]^. Another remarkable taxon found in our dataset was *Xanthomonas*, more specifically *Xanthomonas campestris* (*X. campestris*), which is mostly known as a plant-associated species^[37]^. Murphy *et al.* (2022) have recently reported an increase in the abundance of *X. campestris* following the use of cosmetics products containing Xanthan gum^[38]^. This ingredient is produced by fermentation of monosaccharides by *X. campestris* so that residual DNA might remain present in cosmetic products and consequently could be picked up in our sequencing efforts.

Related to this possible impact of cosmetics, we also performed a more detailed analysis of the association between different lifestyle factors (monitored via a detailed questionnaire) and the relative abundance of the core and other major skin taxa. Significant associations were observed for the number of household members, daily vitamin D supplementation, daily showering or bathing compared to lack of this daily routine, and weekly use of body lotion at the beta diversity level. The association with vitamin D supplements is in line with previous findings that vitamin D deficiency can alter the skin microbiome composition^[20]^. However, to the best of our knowledge, associations between vitamin intake and the skin microbiome have not been described before. We currently do not know whether this change is beneficial, but given the documented role of vitamin D in immune regulation, our data presented here suggest it is of interest to further explore the potential of vitamin D supplementation in inflammatory skin conditions that show a link with a disturbed skin microbiome, such as atopic dermatitis.

Since age plays a crucial role in many inflammatory skin conditions, the correlation between age and the skin microbiome is well documented in previous work^[12,39-42]^. Also in our population, we observed that age was a major factor in defining the skin microbiome composition. Individuals below 12 years old showed significant differences in alpha and beta diversity compared to those aged 12 years and older. In children under 12, we observed a more even distribution in the abundance of the present skin taxa. In individuals of 12 years and older, we observed a higher Inverse Simpson index, where only a limited number of bacterial taxa were more dominant, such as *C. acnes*, but no significant difference was observed for the observed richness. This suggests that the skin microbiome of younger children is still changing to its definite state, where certain taxa will become more dominant. This hypothesis is also substantiated by the finding that the beta diversity showed to be more stable between sampling times in participants of 12 years and older. At species level, we observed a significantly higher abundance of *S. mitis*, *Neisseria*, and *Haemophilus* in children under 12 years old. Their increased presence could be due to the higher rate of nasal secretions (runny noses), colds, and respiratory infections in children^[43,44]^. Notably, in the upper respiratory tract, these taxa seem to be mainly pathogenic^[45]^, while other species such as from the *S. mitis* and *Streptococcus salivarius* group could potentially have a beneficial role in children^[43]^. *C. acnes* and *Cutibacterium avidum* were also more abundant in individuals of 12 years and older. This *C. acnes* increase was previously found on the face of adolescents and adults and is in line with the increased sebum production by the skin at puberty and the ability of *C. acnes* to metabolize sebum components^[13,46]^.

In addition to age, sampling season was shown to be of major importance in our study. Genus richness was found to be significantly greater during the summer sampling period compared to the winter. This is in line with previous work showing that season is an important environmental factor impacting human microbiomes on different human body sites, including skin exhibiting the largest seasonal impact^[47]^. A possible hypothesis for this observation is that the change in season is responsible for this shift in diversity. People typically spend more time outside in the summer and wear less clothes, which might enhance the impact of temperature, humidity, and UV radiation on the microbiome. Specifically for the inner elbow site, there is more exposure of the skin to the environment in the summer when people are wearing short sleeves and increased sweat production in the inner elbow serves as nutrients for skin commensals^[46]^, which could explain the increase in observed diversity. However, to validate this hypothesis, more longitudinal skin microbiome studies should be performed on different skin sites and include more time points, spanning at least two years, to assess whether the microbiome switches back and forth between a personal winter and summer composition. Importantly, we minimized the impact of other factors such as storage conditions or sequencing variables through careful selection of the most appropriate sampling storage conditions for skin metagenomic sequencing, as defined in a previous study^[5]^.

This study has some limitations and strengths. The fact that we only collected two samples per subject and did not sample for multiple years across seasons and life spans are important limitations of our work. Nevertheless, we could already identify several significant associations between individual and lifestyle factors with the elbow skin microbiome, which emphasizes that participant inclusion criteria, such as age and previous conditions, and metadata collection and monitoring of lifestyle, diet and hygiene practices, and the skin microbiome are highly relevant information for future studies. Insights into the longitudinal stability of the healthy skin microbiome are also essential, since many clinical trials run for a certain time period with multiple sampling time points. Because moist skin sites such as the inner elbow are frequently involved in inflammatory skin conditions such as atopic dermatitis, our results are of high value for study designs of future intervention studies to modulate the skin microbiome, e.g., with topical lactobacilli^[19]^. Another important limitation is that samples were collected by the participants themselves, and we had to rely on self-reported evaluation of no skin disorders, which can cause some bias. One reason for this self-reporting and self-sampling at home was that samples were collected during the COVID pandemic (March-June 2021). The influence of this pandemic on the microbiome should, therefore, also be kept in mind (for instance, fewer contacts). Although self-sampling can have several disadvantages, such as cross-contamination of the child’s sample with that of the parent who takes the sample, or too low DNA yields, we could clearly distinguish microbiome profiles based on age, and overall, the samples had good quality. In addition, the research group has experience with self-sampling studies in other human body niches, such as the airways^[48]^, skin^[5]^, and vagina^[35]^. Finally, an important strength of our study is the use of three statistical methods to investigate possible associations of our metadata with the skin microbiome.

In conclusion, the “core skin community” of the inner elbow in our population consists of 14 omnipresent genera and 12 genera with a prevalence of 95% or higher, including several well-known skin bacterial genera, complemented with low abundant and previously unestablished genera that can have a significant impact on the skin microbial community. Several personal, lifestyle, health, hygiene, and environmental factors show significant associations with the skin microbiome composition, the most important of which were age, season, vitamin D supplementation, hygiene habits, and the number of household members. Age was defined as a major factor in shaping the skin microbiome composition and temporal stability, whereas the skin microbiome of children under 12 years old is less stable over a 12-week time period. The substantial seasonal effects observed provide handles towards better synchronization of future clinical skin microbiome studies to limit the seasonal impact on study outputs.

## DECLARATIONS

### Availability of data and materials

Datasets related to this article can be found at https://www.ebi.ac.uk/ena/browser/home, with identification number PRJEB63144, hosted at ENA, EMBL.

### Copyright

© The Author(s) 2024.
