# Supplementary material for: The inner elbow skin microbiome contains Lactobacillus among its core taxa and varies with age, season and lifestyle
Source: Microbiome Res Rep. 2024 Aug 29;3(4):43. doi: 10.20517/mrr.2024.23 (PMC11684916; doi:10.20517/mrr.2024.23)
Supplement: Supplementary file 1 [file mrr-3-4-43-SupplementaryMaterials.pdf]

Delanghe *et al. Microbiome Res Rep* 2024;3:43
DOI: 10.20517/mrr.2024.23

Microbiome Research Reports

Supplementary Materials

The inner elbow skin microbiome contains *Lactobacillus* among its core taxa and varies with age, season and lifestyle

Lize Delanghe[1], Ilke De Boeck[1], Joke Van Malderen[1], Thies Gehrmann[1], Camille Nina Allonsius[1], Peter A. Bron[1], Ingmar Claes[2], Margo Hagendorens[3], Julie Leysen[4], Stijn Wittouck[1], Sarah Lebeer[1]

[1]Department of Bioscience Engineering, University of Antwerp, Antwerpen 2020, Belgium.

[2]YUN NV, Niel 2845, Belgium.

[3]Department of Pediatrics, University Hospital Antwerp/University of Antwerp, Edegem 2650, Belgium.

[4]Department of Dermatology, University Hospital Antwerp/University of Antwerp, Edegem 2650, Belgium.

Correspondence to: Prof. Sarah Lebeer, Department of Bioscience Engineering, University of Antwerp, Groenenborgerlaan 171, Antwerp 2020, Belgium. E-mail: sarah.lebeer@uantwerpen.be

Supplementary Figures

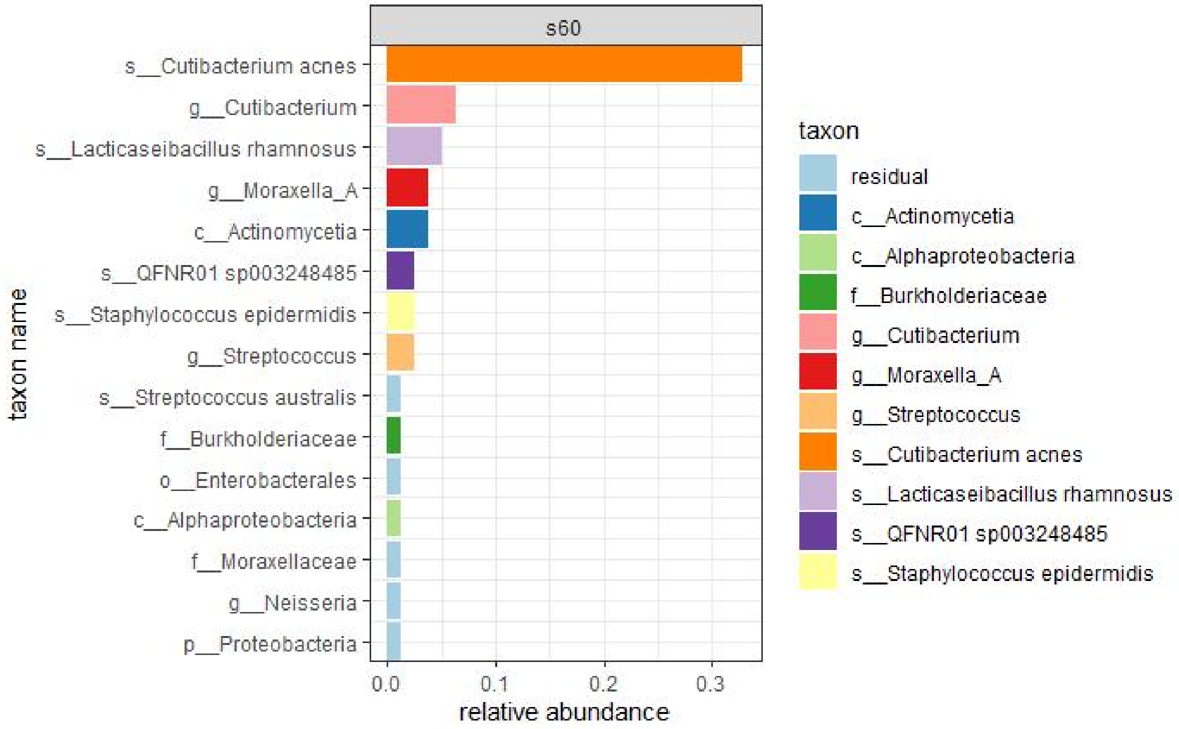

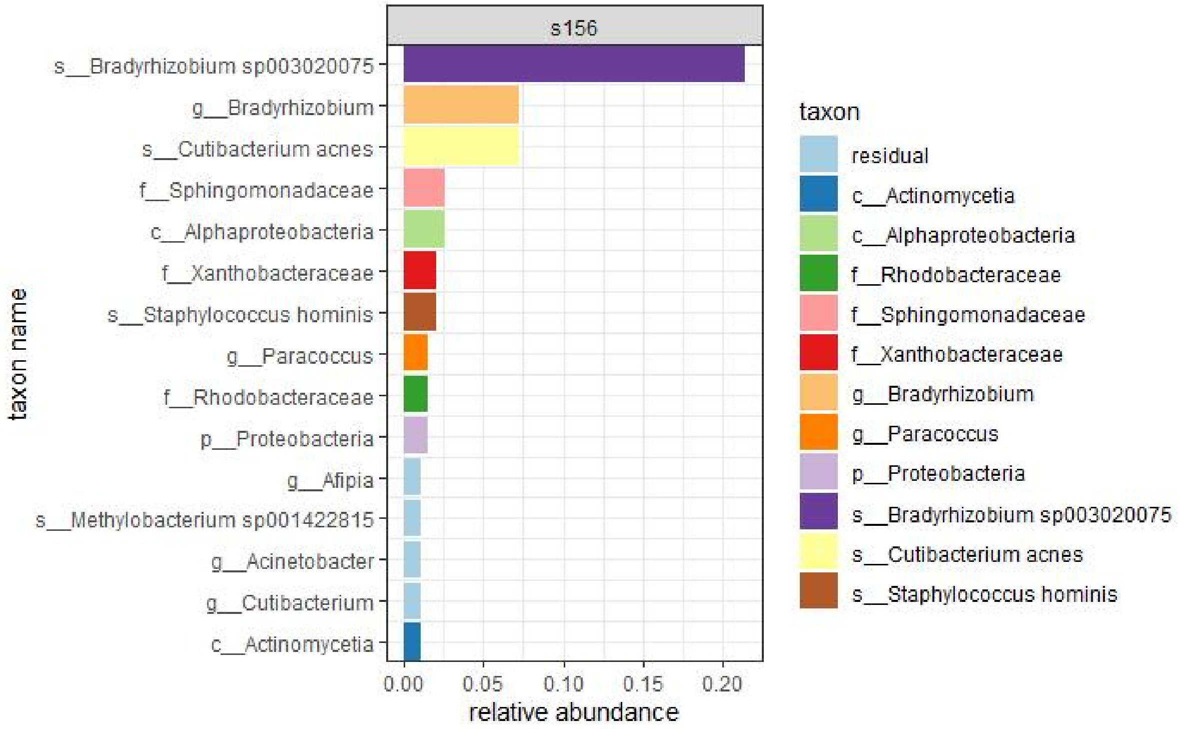

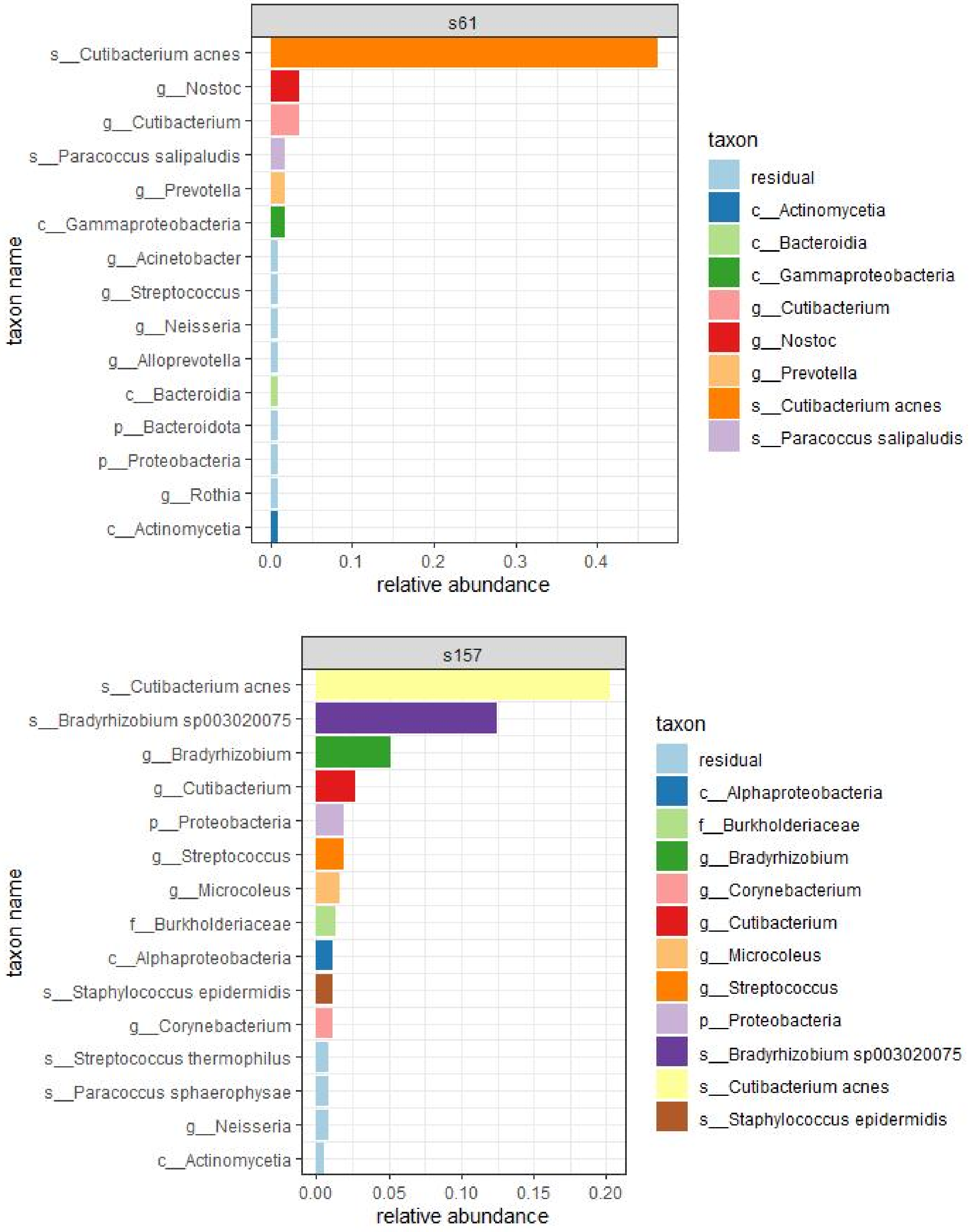

Supplementary Figure 1. Relative taxonomic abundances of negative controls for metagenomic shotgun sequencing.

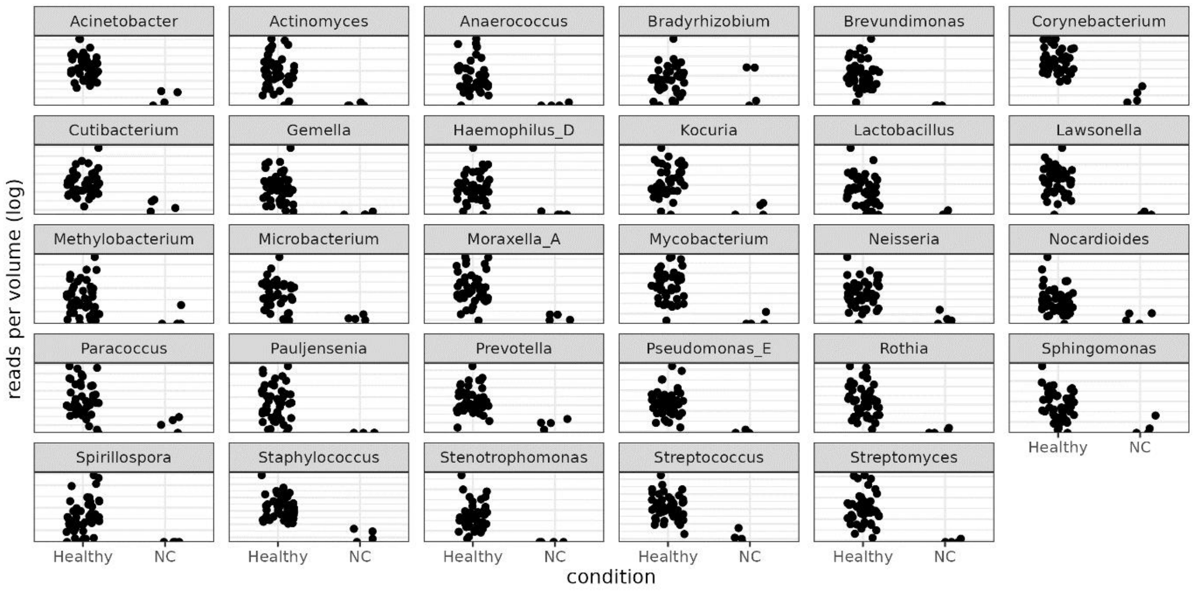

Supplementary Figure 2. Assessment of contaminant genera within the candidate core taxa based on a prevalence of at least 95%in the T0 samples.

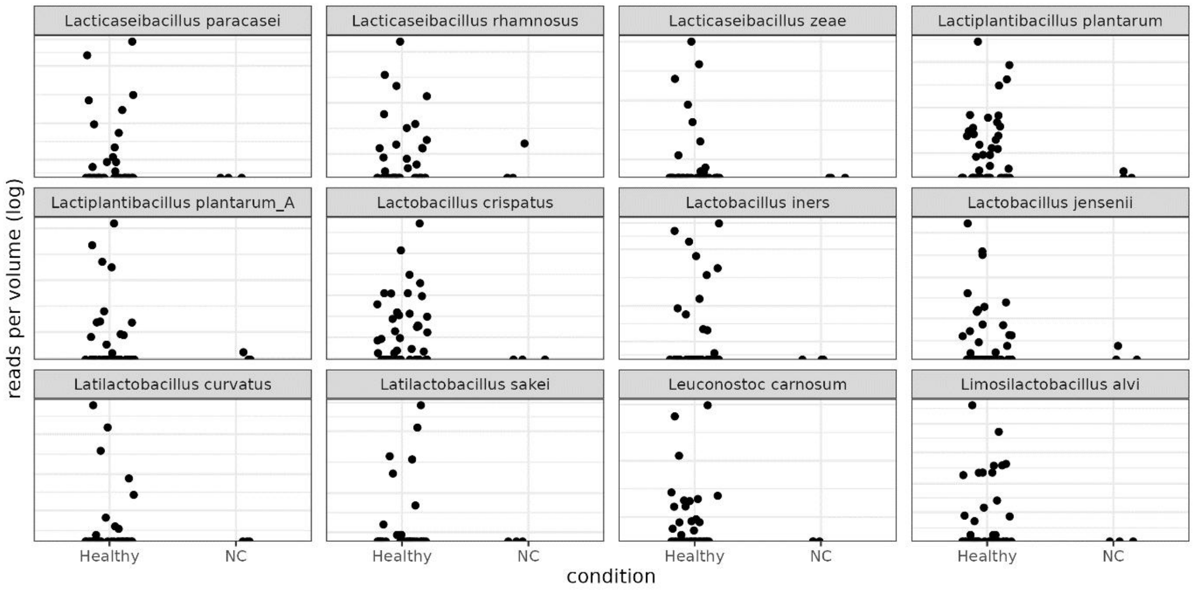

Supplementary Figure 3. Assessment of contaminant *Lactobacillaceae* based on a prevalence of at least 20% in the T0 samples.

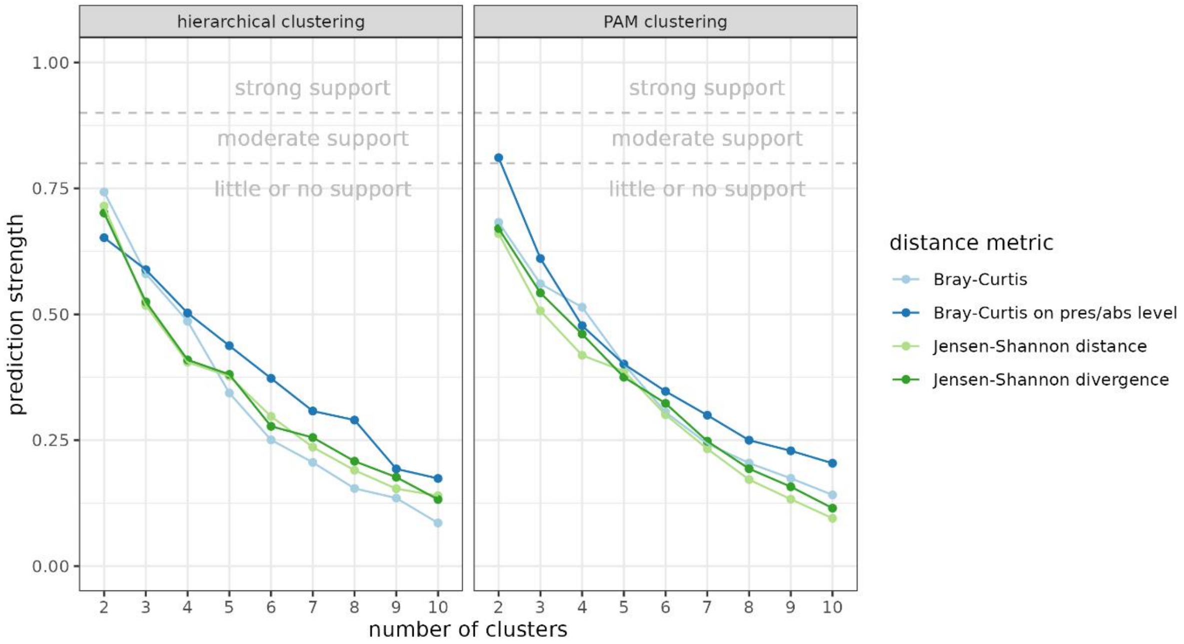

Supplementary Figure 4. Prediction strength to confirm absence of specific clusters in our sample cohort for both hierarchical (left) and PAM clustering (right). Prediction strength was measured based on four distance matrices: Bray-Curtis (on relative abundances), Bray-Curtis on the presence/absence level, Jensen-Shannon divergence and Jense-Shannon distance (equal to the square root of the Jensen-Shannon divergence). For hierarchical clustering, moderate support was only observed for one distance matrix. For PAM clustering, no support was observed for significant clusters in our dataset.

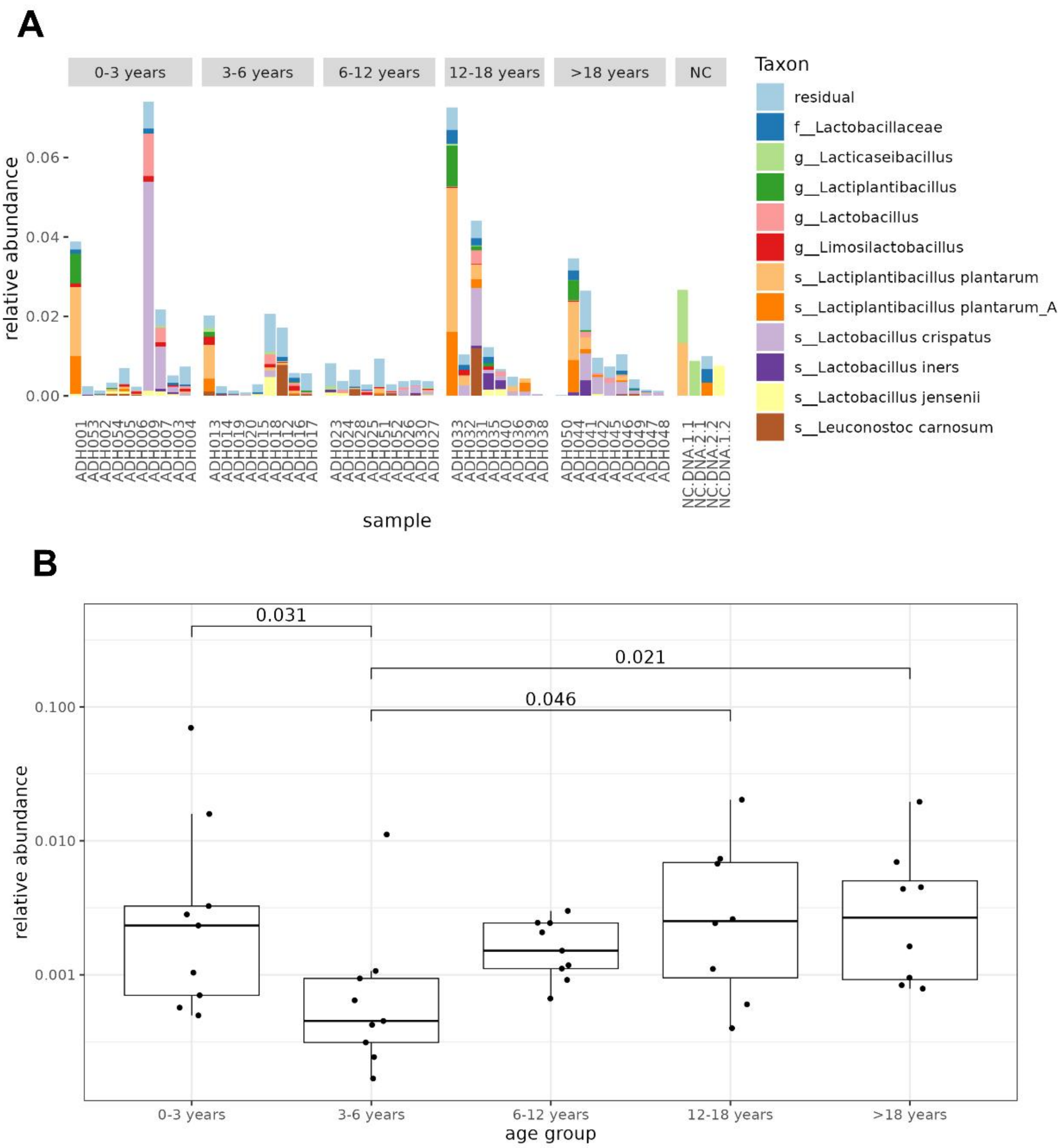

Supplementary Figure 5. (A) Bar chart presenting the relative abundance of members of the *Lactobacillaceae* family in all samples, grouped in age groups and controls (at baseline); (B) Boxplot comparing the relative abundance of the *Lactobacillus* genus between the different age groups. Statistical differences between the groups were tested using Wilcoxon rank sum test, which is based on the ranking of the samples.

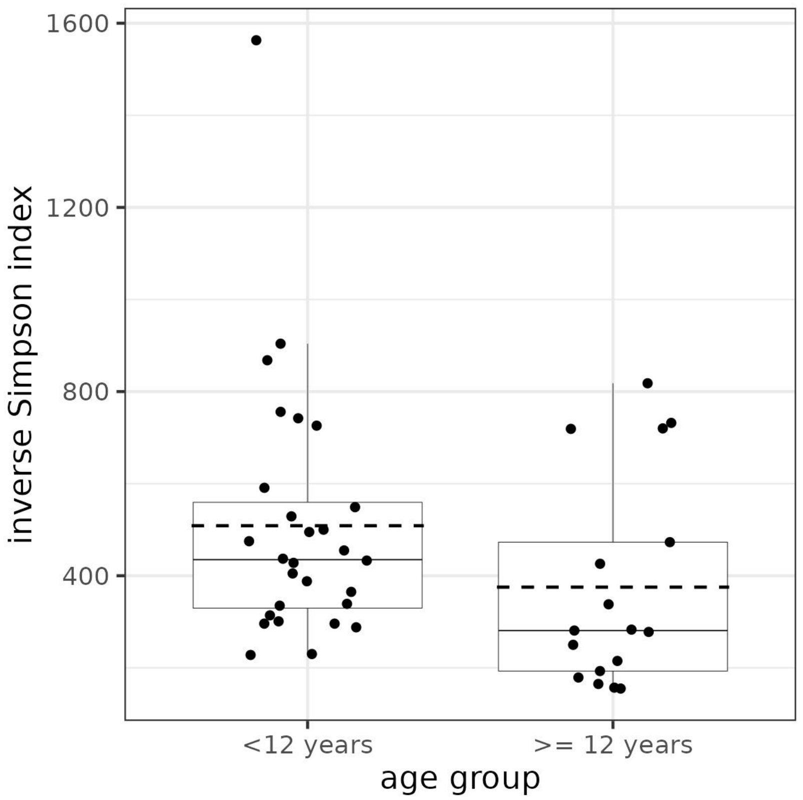

Supplementary Figure 6. Observed richness for skin swabs of healthy people below 12 years old and from 12 years and older (at baseline), at genus level. Dashed lines represent the mean Inverse Simpson index.

Supplementary Tables

Supplementary Table 1. Participants' demographics and information

**Total (*n* = 52)**		
	**[n]**	**[%]**
Age (years)		
- Range	0 – 53	/
- Mean, stdv	11.87 +/- 11.83 *	/
- 0-3 years	11	21.15
- 3-6 years	10	19.23
- 6-12 years	11	21.15
- 12-18 years	10	19.23
- >18 years	10	19.23
Oral antibiotics in last 3 months	3	5.77
Allergy	4	7.69
Close family members with allergy	21	40.38
Close family members with AD	5	9.62
Probiotic supplements more than 3 times per week	5	9.62
Probiotic dairy products weekly	3	5.77
Fermented food products last week	38	70.08
Dairy products daily	31	59.62
Vitamin D supplements daily	19	36.54
- 0-3 years	7	58.33
- 3-6 years	5	50
- 6-12 years	5	41.67
- 12-18 years	1	10
- >18 years	1	10
Antibacterial soap daily	15	28.84
Daily shower or bath	12	23.08
Use of scrub weekly	9	17.31
Use of bodylotion weekly	14	26.92
More than 2 hours a day outdoors	21	40.38
Frequent contact with animals	27	51.92
Pet cat	15	28.84
Pet dog	8	15.38
More than 4 house members	17	32.69

*Age at the first sampling timepoint

Supplementary Table 2. DNA concentrations, start volumes and library sizes of the samples used for shotgun sequencing

Run	Sample ID	Start concentration Qubit (ng/µL)	Start volume for library prep (µL)	Qubit (ng/µL) (after PCR)	Library size (bp)	nM	ul sample for final 4 nM pool
1	ADH001_T0_DNA	TL (≤ 0.05)	30	1,98	233	12,87553648	8,4
1	ADH001_T12_DNA	TL	30	21,5	393	82,88996839	1,304796087
1	ADH002_T0_DNA	TL	30	1,75	190	13,9553429	7,750042918
1	ADH002_T12_DNA	TL	30	14,7	272	81,88502674	1,320809319
1	ADH003_T0_DNA	TL	30	0,771	188	6,213733075	15,7
1	ADH003_T12_DNA	TL	30	14,2	290	74,19017764	1,457800883
1	ADH004_T0_DNA	TL	30	7,93	233	51,56717388	2,097351828
1	ADH004_T12_DNA	TL	30	38,3	318	182,4852297	0,5926754
1	ADH005_T0_DNA	TL	30	2,56	234	16,57601658	6,524758584
1	ADH005_T12_DNA	TL	30	10,2	277	55,79258287	1,938510477
1	ADH006_T0_DNA	TL	30	1,2	220	8,26446281	11
1	ADH006_T12_DNA	TL	30	9,06	256	53,62215909	2,016974107
1	ADH007_T0_DNA	TL	30	0,752	169	6,741975973	16,04195964
1	ADH007_T12_DNA	TL	30	50	350	216,4502165	0,49967382
1	ADH008_T0_DNA	TL	30	1,28	196	9,894867038	10,93036481
1	ADH008_T12_DNA	TL	30	50	357	212,2060946	0,509667296
1	ADH009_T0_DNA	TL	30	5,43	237	34,71423092	3,115566832
1	ADH009_T12_DNA	TL	30	26,1	312	126,7482517	0,853301761
1	ADH010_T0_DNA	TL	30	0,35	117	4,532504533	20,5
1	ADH010_T12_DNA	TL	30	38,1	340	169,7860963	0,637004495
1	ADH011_T0_DNA	TL	30	0,246	135	2,760942761	20
1	ADH011_T12_DNA	TL	30	41,1	422	147,5657044	0,732924407
1	ADH012_T0_DNA	TL	30	3,39	247	20,79499448	5,200987504
1	ADH012_T12_DNA	TL	30	23,6	391	91,4516004	1,182642031
1	ADH013_T0_DNA	TL	30	0,86	144	9,048821549	11,95233057
1	ADH013_T12_DNA	TL	30	37,5	297	191,3070095	0,565345236
1	ADH014_T0_DNA	TL	30	30,5	387	119,4111659	0,905731936

1	ADH014_T12_DNA	TL	30	33,6	420	121,2121212	0,892274678
1	ADH015_T0_DNA	TL	30	0,956	212	6,8324757	15,82947546
1	ADH015_T12_DNA	TL	30	13,8	252	82,97258297	1,303496921
1	ADH016_T0_DNA	TL	30	2,65	244	16,455539	6,57252895
1	ADH016_T12_DNA	TL	30	35,8	488	111,1525087	0,973028029
1	ADH017_T0_DNA	TL	30	24,6	281	132,6431576	0,815379462
1	ADH017_T12_DNA	TL	30	32,7	475	104,3062201	1,036894121
1	ADH018_T0_DNA	TL	30	1,12	171	9,923799397	10,89849785
1	ADH018_T12_DNA	TL	30	11,8	235	76,07994842	1,421590165
1	ADH019_T0_DNA	TL	30	12,7	297	64,78930721	1,669326484
1	ADH019_T12_DNA	TL	30	39,2	453	131,112449	0,824898835
1	ADH020_T0_DNA	TL	30	11,5	266	65,50467077	1,6510961
1	ADH020_T12_DNA	TL	30	45,4	376	182,9464861	0,591181108
1	ADH021_T0_DNA	TL	30	0,772	203	5,762054038	18,77013053
1	ADH021_T12_DNA	TL	30	5,12	279	27,80493103	3,889759925
1	ADH022_T0_DNA	TL	30	9,38	276	51,4931928	2,100365127
1	ADH022_T12_DNA	TL	30	18,1	280	97,94372294	1,104251535
1	ADH023_T0_DNA	TL	30	1,38	228	9,170653907	11,79354357
1	ADH023_T12_DNA	TL	30	27,8	284	148,3141272	0,729225924
1	ADH024_T0_DNA	TL	30	0,686	183	5,679748303	19,04213016
1	ADH024_T12_DNA	TL	30	19,1	252	114,8388648	0,941793587
1	ADH025_T0_DNA	TL	30	2,1	212	15,00857633	7,206180258
1	ADH026_T0_DNA	TL	30	4,14	244	25,70789866	4,207053555
1	ADH026_T12_DNA	TL	30	23,2	349	100,7206738	1,073806423
1	ADH027_T0_DNA	TL	30	0,979	222	6,681681682	16,18671939
1	ADH027_T12_DNA	TL	30	22	308	108,2251082	0,999347639
1	NC_DNA_1	TL	30	0,279	98	4,313543599	25,07323827
1	NC_DNA_2	TL	30	0,308	86	5,426356589	19,93133047
2	ADH028_T0_DNA	TL (≤ 0.05)	30	0,7	177	5,992124636	24
2	ADH028_T12_DNA	TL	30	18,7	300	94,44444444	1,522704613

2	ADH030_T0_DNA	TL	30	0,779	172	6,86222692	20,95689824
2	ADH030_T12_DNA	TL	30	21,9	287	115,6160912	1,243866574
2	ADH031_T0_DNA	TL	30	1,74	259	10,17901018	14,12819014
2	ADH031_T12_DNA	TL	30	2,35	239	14,89793331	9,653083303
2	ADH032_T0_DNA	TL	30	0,498	136	5,548128342	24
2	ADH032_T12_DNA	TL	30	5,72	304	28,50877193	5,044447078
2	ADH033_T0_DNA	TL	30	1,48	201	11,15633951	12,89051764
2	ADH033_T12_DNA	TL	30	5,59	253	33,47706312	4,295806677
2	ADH034_T0_DNA	TL	30	0,382	104	5,565268065	24
2	ADH034_T12_DNA	TL	30	3,54	264	20,31680441	7,07842574
2	ADH035_T0_DNA	TL	30	0,864	219	5,97758406	24,05838041
2	ADH035_T12_DNA	TL	30	7,17	240	45,26515152	22,28
2	ADH036_T0_DNA	TL	30	0,682	137	7,542579075	19,066554
2	ADH036_T12_DNA	TL	30	2,93	233	19,05319287	7,54786834
2	ADH037_T0_DNA	TL	30	0,198	148	2,027027027	24
2	ADH037_T12_DNA	TL	30	3,34	234	21,62652163	6,649751345
2	ADH038_T0_DNA	TL	30	0,504	154	4,958677686	24
2	ADH038_T12_DNA	TL	30	10,1	235	65,11927789	2,208424232
2	ADH039_T0_DNA	TL	30	0,437	166	3,988682001	22
2	ADH039_T12_DNA	TL	30	6,91	251	41,71194012	3,447717629
2	ADH040_T0_DNA	TL	30	0,57	158	5,466052934	22
2	ADH040_T12_DNA	TL	30	18,7	242	117,0798898	1,228315055
2	ADH041_T0_DNA	TL	30	0,477	173	4,177614293	23
2	ADH041_T12_DNA	TL	30	3,76	233	24,45051372	5,881716552
2	ADH042_T0_DNA	TL	30	0,937	252	5,633718134	25,52683465
2	ADH042_T12_DNA	TL	30	54	287	285,0807729	0,504456999
2	ADH043_T0_DNA	TL	30	8,36	261	48,53128991	2,96326332
2	ADH043_T12_DNA	TL	30	9,55	279	51,86271315	2,772916852
2	ADH044_T0_DNA	TL	30	0,871	208	6,34469697	21
2	ADH044_T12_DNA	TL	30	9,91	245	61,28633271	2,346542612

2	ADH045_T0_DNA	TL	30	0,456	146	4,732254047	22
2	ADH045_T12_DNA	TL	30	3,47	244	21,54744163	6,674156206
2	ADH046_T0_DNA	TL	30	0,57	173	4,992117709	25
2	ADH046_T12_DNA	TL	30	29,5	312	143,2595183	1,003849469
2	ADH047_T0_DNA	TL	30	1,35	248	8,247800587	17,43628374
2	ADH047_T12_DNA	TL	30	24,3	290	126,9592476	1,132733487
2	ADH048_T0_DNA	TL	30	0,461	146	4,784142798	23
2	ADH048_T12_DNA	TL	30	9,02	267	51,18601748	2,809575707
2	ADH049_T0_DNA	TL	30	0,639	313	3,093232646	22
2	ADH049_T12_DNA	TL	30	9,55	285	50,77086656	2,832549472
2	ADH050_T0_DNA	TL	30	0,404	166	3,687477181	24
2	ADH050_T12_DNA	TL	30	19,4	389	75,56282621	1,903197624
2	ADH051_T0_DNA	TL	30	1,61	211	11,56110872	12,43920413
2	ADH051_T12_DNA	TL	30	43,6	296	223,1777232	0,64437879
2	ADH052_T0_DNA	TL	30	0,898	198	6,871747781	20,92786229
2	ADH052_T12_DNA	TL	30	14,9	278	81,20776106	1,770902059
2	ADH053_T0_DNA	TL	30	1,73	208	12,60198135	11,41177623
2	ADH053_T12_DNA	TL	30	33,6	387	131,5480385	1,093220339
2	ADH054_T0_DNA	TL	30	2,32	249	14,11707436	10,18702513
2	ADH054_T12_DNA	TL	30	38,5	384	151,9097222	0,946687211
2	NC_DNA_1	TL	30	0,343	122	4,259811227	21
2	NC_DNA_2	TL	30	0,526	178	4,477357848	24,5

Supplementary Table 3. Read counts for all included skin samples after quality control. NC = negative control. T0 = baseline (winter sample), T12 = week 12 (summer sample)

Sample ID	Bacterial read counts	Total read counts
ADH001.T0	2036	67724
ADH001.T12	22794	170938
ADH002.T0	4961	40338
ADH002.T12	5338	83345
ADH003.T0	2171	20628
ADH003.T12	12156	78385
ADH004.T0	2843	60835
ADH004.T12	13225	177040
ADH005.T0	1725	74817
ADH005.T12	19138	122771
ADH006.T0	3960	26191
ADH006.T12	2771	59784
ADH007.T0	1724	13866
ADH007.T12	21391	389844
ADH009.T0	3099	64854
ADH009.T12	26243	157344
ADH010.T12	42322	141295
ADH011.T12	50768	288104
ADH012.T0	4152	70958
ADH012.T12	40718	222537
ADH013.T0	950	12141
ADH013.T12	10976	118011
ADH014.T0	6650	158910
ADH014.T12	26819	287366
ADH015.T0	2385	44060
ADH015.T12	5838	76380
ADH016.T0	3119	83659
ADH016.T12	13662	434653
ADH017.T0	2138	135523

ADH017.T12	9948	407268
ADH018.T0	1267	18563
ADH018.T12	1639	69223
ADH019.T0	12160	73818
ADH019.T12	17740	356334
ADH020.T0	12837	99372
ADH020.T12	55719	229520
ADH023.T0	1244	35786
ADH023.T12	10031	136359
ADH024.T0	1330	23524
ADH024.T12	11490	126711
ADH025.T0	4539	29986
ADH026.T0	9233	56615
ADH026.T12	22541	149161
ADH027.T0	6052	28590
ADH027.T12	16152	107841
NC.DNA.1.1	79	166
NC.DNA.2.1	114	198
ADH028.T0	3050	34249
ADH028.T12	35166	139003
ADH030.T0	4136	28663
ADH030.T12	14573	82165
ADH031.T0	11565	86977
ADH031.T12	14654	43213
ADH032.T0	773	4238
ADH032.T12	14755	94236
ADH033.T0	10215	43087
ADH033.T12	22444	94255
ADH034.T12	31834	86142
ADH035.T0	1236	43207
ADH035.T12	41048	563391
ADH036.T0	827	3913

ADH036.T12	14322	92389
ADH037.T12	11912	84419
ADH038.T0	2510	4619
ADH038.T12	18894	77799
ADH039.T0	908	3663
ADH039.T12	16566	83412
ADH040.T0	1793	6328
ADH040.T12	33240	117440
ADH041.T0	2062	8247
ADH041.T12	15564	84781
ADH042.T0	2310	42600
ADH042.T12	12832	143817
ADH044.T0	12417	47137
ADH044.T12	25740	93432
ADH045.T0	1781	10332
ADH045.T12	101318	361869
ADH046.T0	9149	15892
ADH046.T12	23653	57830
ADH047.T0	60978	92618
ADH047.T12	91692	168605
ADH048.T0	3162	8745
ADH048.T12	54010	171644
ADH049.T0	10772	17671
ADH049.T12	2884	9881
ADH050.T0	4807	6057
ADH050.T12	127632	244515
ADH051.T0	2685	74437
ADH051.T12	26889	180086
ADH052.T0	5499	49025
ADH052.T12	32350	164769
ADH053.T0	12864	63012
ADH053.T12	91081	367823

ADH054.T0	3537	106686
ADH054.T12	44577	336476
NC.DNA.1.2	192	399
NC.DNA.2.2	370	1201

Supplementary Table 4. Overview of 15 taxa with highest mean relative abundances and prevalences

Species	Mean relative abundance	Prevalence
Cutibacterium acnes	0.14427320033147736	1
Staphylococcus epidermidis	0.013431339321984401	1
Cutibacterium namnetense	0.005770237834602315	1
Staphylococcus hominis	0.06253535861973401	0.9777777777777777
Moraxella_A osloensis	0.009622031031301067	0.9777777777777777
Moraxella_A sp002478835	0.007389508657136736	0.9777777777777777
Mycobacterium sp001665295	0.007301113899830347	0.9777777777777777
Lawsonella clevelandensis_A	0.009979509601845318	0.9555555555555556
Staphylococcus capitis	0.02364257883056313	0.9333333333333333
Acinetobacter johnsonii	0.00574408604739489	0.9111111111111111
Micrococcus luteus	0.014115880435083902	0.8888888888888888
Moraxella_A cinereus	0.00823660384569195	0.8888888888888888
Kocuria rhizophila	0.005966598190028093	0.8444444444444444
Corynebacterium kroppenstedtii_B	0.003515656276879128	0.8222222222222222
Xanthomonas campestris	0.005452592230116739	0.7333333333333333

Supplementary Questionnaires

Questionnaire T0 – children

Q2 Gender

▼ Woman (1) ... Other (3)

Birth year

▼ 2002 (121) ... 2021 (139)

Length (in cm)

▼ 0 (1) ... 250 (251)

Weight (in kg)

▼ 0 (1) ... 199 (398)

What is the country of birth of the child?

▼ Afghanistan (1) ... Zimbabwe (1357)

With how many people does your child live with? If you have multiple main residences (e.g. in case of co-parenting, at boarding school, in a student room, etc.), please indicate the sum of all residences:

1. 0 (1)
2. 1 (2)
3. 2 (3)
4. 3 (4)
5. 4 (5)
6. 5 (6)
7. 6 (7)
8. 7 (8)
9. 8 (9)
10. 9 (10)
11. 10 (11)
12. > 10 (12) ____

How many siblings does your child has?

13. 0 (1)
14. 1 (2)
15. 2 (3)
16. 3 (4)
17. 4 (5)
18. 5 (6)
19. 6 (7)
20. 7 (8)
21. 8 (9)
22. 9 (10)
23. 10 (11)
24. > 10 (12) ____

Ethnicity of the child?

25. White (1)
26. Black African (2)
27. North African (3)
28. South American (4)
29. Asian (5)
30. Other: (6) ____

Does your child smoke?

31. Yes, how many cigarettes each day? (1)
    ____
32. No, ex-smoker since: (2) ____
33. No (3)

Is your child exposed to smoking in his/her direct environment (indoors, car, …)

34. Yes (1)
35. No (2)
36.

With the following questions we want to know the main activities of your child:

Is your child going to daycare?

37. Yes (1)
38. No (2)

Is your child going to school?

39. Yes (1)
40. No (2)

If yes,

41. Kindergarten (1)
42. Primary school (2)
43. Secundary education (3)
44. Higher education (4)
45. Other: (5) ____

If not, how does your child receive education?

46. Home school (1)
47. Other: (2) ____

Does your child have contact with animals during school or free time (several answers are possible)?

- Yes, at school/work, which animals? (1) ____
- Yes, I have pets, how many and which ones? (2) ____
- No (3)
- Other: (4) ____

Approximately how many hours per day does your child spend outside?

▼ 0 (1) ... 24 (25)

Personal hygiene:

	Multiple times a day (1)	Daily (2)	> 3x/week (3)	Weekly (4)	Monthly (5)	Seldom (6)	Never (7)
How often does your child shower(1)	48.	49.	50.	51.	52.	53.	54.
How often does your child take a bath (2)	55.	56.	57.	58.	59.	60.	61.
How often does your child use body lotion/milk? (3)	62.	63.	64.	65.	66.	67.	68.
How often does your child use soap when washing (body)? (4)	69.	70.	71.	72.	73.	74.	75.
How often does your child use skin peels or scrubs (5)	76.	77.	78.	79.	80.	81.	82.
How often does your child use antibacterial soap or hand disinfectants (e.g. Dettol, disinfectant hand gel, Stellisept,...? (6)	83.	84.	85.	86.	87.	88.	89.
How often does your child use parfum (8)	90.	91.	92.	93.	94.	95.	96.

Dietary pattern:

How often does your child use probiotics or "good bacteria" in the products below?

	Never (1)	< 3x per week (2)	> 3x per week (3)	Daily (4)
In dairy products (bv. Kefir, Yakult, Actimel etc.) (1)	97.	98.	99.	100.
In capsules such as Enterol, Probactiol or other supplements available at the pharmacy (2)	101.	102.	103.	104.

If yes:

- Which products? (1) ____
- Has your child used such products in the past week? (Yes/No) (2)

  ____

Does your child regularly consume dairy products (cheese, milk, yogurt, etc.)?

105. Multiple times per day (1)
106. Daily (2)
107. A few times per week (3)
108. Weekly (4)
109. Seldom (5)
110. Never (6)
111.

Has your child used such products in the past week?

112. Yes (1)
113. No (2)
114.

Does your child regularly consume fermented foods (olives, salami, sauerkraut, sourdough bread, pickles, yogurt, milk, buttermilk, kefir, chocolate, soy sauce, tofu, etc.)?

115. Multiple times per day (1)
116. Daily (2)
117. A few times per week (3)
118. Weekly (4)
119. Seldom (5)
120. Never (6)

Has your child used such products in the past week?

121. Yes (1)
122. No (2)

Does your child take vitamins, minerals or other supplements?

123. Yes: How often and which ones? (1)

____

124. No (2)

Has your child food intolerances?

125. Yes (1)
126. No (2)

If yes, which ones (multiple answers possible)

- Lactose intolerance (1)
- Gluten intolerance (2)
- Other: (3) ____

Medical information:

How would you describe the general health of your child?

127. Very good (1)
128. Good (2)
129. Moderate (3)
130. Bad (4)
131. Very bad (5)
132. Oher: (6) ____

Is your child receiving any antibiotics at the moment?

133. Yes (4)
134. No (5)
135.

When was your child's last antibiotic treatment, both oral (by mouth) and topical (locally, usually in the form of a cream) (approximately)?

____

Has your child atopic eczema?

136. Yes (1)
137. No (2)

Does your child have another skin condition (besides atopic eczema)?

138. Yes, which one(s)? (1) ____
139. No (2)

Has your child previously had atopic eczema or another skin condition?

140. Yes (1)
141. No (2)

If yes, which condition?

____

If yes, when did the last symptoms occur (year)?

____

Does your child suffer from asthma?

142. Yes, since (year): (1) ____
143. No (2)

If so, is your child taking medication to treat asthma?

144. Yes: (name of the product) (1) ____
145. No (2)

Does your child have allergies?

146. Yes (1)
147. No (2)

Does your child take antihistamines (to treat allergies, itching due to eczema, etc.)?

148. Yes (1)
149. No (2)

Does your child suffer from certain diseases (several answers are possible)? (You do not need to fill in information regarding asthma and/or atopic eczema here)

- Skin: (1) ____
- Muscles/joints: (2) ____
- Heart/blood vessels: (3) ____
- Urinary tract: (4) ____
- Stomach/intestines: (5) ____
- Respiratory tract: (6) ____
- Nerve system: (7) ____
- Eyes: (8) ____
- Ear-nose-throat: (9) ____
- Hormonal (thyroid, diabetes, …): (10) ____
- Blood: (11) ____
- Cancer (treated with chemotherapy, radiotherapy, hormone treatment or other): (12) ____
- Immune disease: (13) ____
- Other: (14) ____
- No (15)

Do any family members suffer from atopic eczema, another skin condition, asthma or allergies?

150. Yes (1)
151. No (2)

If so, what relationship do you have with these people? What conditions do these people suffer from?

	Relationship with this person (daughter, son, niece, nephew, aunt, uncle, ...) (1)	Which disease? (which allergy, skin condition, asthma, ...) (2)
Person 1 (1)		
Person 2 (4)		
Person 3 (5)		

Person 4 (6)		
Person 5 (7)		
Person 6 (8)		

Did your child use medication in the past 3 months?

152. Yes (1)
153. No (2)

If your child had to take medication in the past 3 months, please enter this in the text box below. Please list the name, start and stop date and reason for each product.

	Name (1)	Dose (2)	Start date (3)	Stop date (4)	Reason (5)
Medication 1 (3)					
Medication 2 (4)					
Medication 3 (5)					

You have reached the end of this questionnaire. Thanks for completing!

Other relevant notes:

____

____

Questionnaire T0 – Adults

Q2 Gender

▼ Woman (1) ... Other (3)

Q3 Birth year

▼ 1920 (1) ... 2003 (2212)

Q4 Length (in cm)

▼ 0 (1) ... 250 (251)

Q99 Weight (in kg)

▼ 0 (1) ... 199 (200)

Q94 Country of birth:

▼ Afghanistan (1) ... Zimbabwe (1357)

Q6: How many people do you live with? If there are multiple main places of residence (e.g. co-parenting, in a student room, at boarding school, etc.), please indicate the sum of all places of residence:

- ○ 0 (1)
- ○ 1 (2)
- ○ 2 (3)
- ○ 3 (4)
- ○ 4 (5)
- ○ 5 (6)
- ○ 6 (7)
- ○ 7 (8)
- ○ 8 (9)
- ○ 9 (10)
- ○ 10 (11)
- ○ > 10: (12) ____

Q7 How many siblings do you have?

- ○ 0 (1)
- ○ 1 (2)
- ○ 2 (3)
- ○ 3 (4)
- ○ 4 (5)
- ○ 5 (6)
- ○ 6 (7)
- ○ 7 (8)
- ○ 8 (9)
- ○ 9 (10)
- ○ 10 (11)
- ○ >10: (12) ____

Q8 Ethnicity

- ○ White (1)
- ○ Black African (2)
- ○ North African (3)
- ○ South American (4)
- ○ Asian (5)
- ○ Other: (6) ____

Q9 Do you smoke?

- ◯ Yes, how many cigarettes each day? (1) ____
- ◯ No, ex-smoker since: (2) ____
- ◯ No (3)

Q10 Are you exposed to smoke in your direct environment (indoors/car)?

- ◯ Yes (1)
- ◯ No (2)

Q101 Do you use drugs

- ◯ Yes, which ones? (1) ____
- ◯ No, ex-drug user since (year): (2) ____
- ◯ No (3)

Q13 Are you going to work outside your home?

- ◯ Yes (1)
- ◯ No (2)

Q83 What is your job description?

____

Q25 Do you have contact with animals at work or in your free time?

- ☐ Yes, at work, which animals (1) ____
- ☐ Yes, I have pets, how many and which ones? (2) ____
- ☐ No (3)
- ☐ Other: (4) ____

Q27 Approximately how many hours per day do you spend outside?

▼ 0 (1) ... 24 (25)

Q28 Personal hygiene:

	Multiple times a day (1)	Daily (2)	> 3x/week (3)	Weekly (4)	Monthly (5)	Seldom (6)	Never (7)
How often do you shower(1)	154.	155.	156.	157.	158.	159.	160.
How often do you take a bath (2)	161.	162.	163.	164.	165.	166.	167.
How often do you use body lotion/milk? (3)	168.	169.	170.	171.	172.	173.	174.
How often do you use soap when washing (body)? (4)	175.	176.	177.	178.	179.	180.	181.
How often do you use skin peels or scrubs (5)	182.	183.	184.	185.	186.	187.	188.
How often do you use antibacterial soap or hand disinfectants (e.g. Dettol, disinfectant hand gel, Stellisept,...? (6)	189.	190.	191.	192.	193.	194.	195.
How often do you use parfum (8)	196.	197.	198.	199.	200.	201.	202.

Q86 Dietary pattern:

Q95 How often do you use probiotics or “ good bacteria” in the products below?

	Never (1)	< 3x per week (2)	> 3x per week (3)	Daily (4)
In dairy products (bv. Kefir, Yakult, Actimel etc.) (1)	203.	204.	205.	206.
In capsules such as Enterol, Probactiol or other supplements available at the pharmacy (2)	207.	208.	209.	210.

Q79 If yes:

- ▢ Which products? (1) ____
- ▢ Did you consume such products in the past week? (yes/no) (3) ____

Q31 DO you regulalry consume dairy products (cheese, milk, yoghurt, …)?

- o Multiple times per day (1)
- o Daily (2)
- o A few times per week (3)
- o Weekly (4)
- o Seldom (5)
- o Never (6)

Q32 Did you consume such products in the past week?

- ○ Yes (1)
- ○ No (2)

Q33 Do you regularly consume fermented foods (olives, salami, sauerkraut, sourdough bread, pickles, yogurt, milk, buttermilk, kefir, chocolate, soy sauce, tofu, etc.)?

- ○ Multiple times per day (1)
- ○ Daily (2)
- ○ A few times per week (3)
- ○ Weekly (4)
- ○ Seldom (5)
- ○ Never (6)

Q35 Did you consume such products in the past week?

○ Yes (1)

○ No (2)

Q42 Do you take vitamins, minerals or other supplements?

○ Yes: how often and which ones? (1)
____

○ No (2)

Q43 Do you have food intolerances?

○ Yes (1)

○ No (2)

Q44 If yes, which one (multiple answers possible)

☐ Lactose-intolerance (1)

☐ Gluten intolerance (2)

☐ Other: (3) ____

Q85 Medical information:

Q45 How would you describe your general health?

- Very good (1)
- Good (2)
- Average (3)
- Bad (4)
- Very bad (5)
- Other: (6) ____

Q88 Are you currently taking any antibiotics?

- ◯ Yes (1)
- ◯ No (2)

Q47 When was your last antibiotic treatment, both oral (by mouth) and topical (locally, usually in the form of a cream) (approximately)?

____

Q102 Do youhave atopic eczema?

- ◯ Yes, since (year): (1) ____
- ◯ No (2)

Q103 Do you have other skin conditions?

- ◯ Yes, which ones? (1) ____
- ◯ No (2)

Q104 Have you previously had atopic eczema or another skin condition?

- ◯ Yes (1)
- ◯ No (2)

Q105 If yes, which condition?

____

Q106 If yes, when did you experience the last symptoms?

____

Q48 Do you suffer from asthma?

- ○ Yes, since (year) (1) ____
- ○ No (2)

Q49 If yes, do you take medication for your asthma?

- ○ Yes: (name of the product) (1) ____
- ○ No (3)

Q50 Do you have allergies? (multiple answers possible)

- ☐ Hay fever/pollen (1)
- ☐ House dust mite (2)
- ☐ Latex (3)
- ☐ Animals, which one? (4) ____
- ☐ Food allergy, which one? (5) ____
- ☐ Other: (6) ____
- ☐ No allergies (7)

Q110 Do you take antihistamines?

- ○ Yes (1)
- ○ No (2)

Q55 Do you suffer from certain diseases (several answers are possible)? (You do not need to fill in information regarding asthma and/or atopic eczema here)

- Skin: (1) ____
- Muscles/joints: (2) ____
- Heart/blood vessels: (3) ____
- Urinary tract: (4) ____
- Stomach/intestines: (5) ____
- Respiratory tract: (6) ____
- Nerve system: (7) ____
- Eyes: (8) ____
- Ear-nose-throat: (9) ____
- Hormonal (thyroid, diabetes, …): (10) ____
- Blood: (11) ____
- Cancer (treated with chemotherapy, radiotherapy, hormone treatment or other): (12) ____
- Immune disease: (13) ____
- Other: (14) ____
- No (15)

Q108 Do any family members suffer from atopic eczema, another skin condition, asthma or allergies?

○ Yes (1)

○ No (2)

Q109 If so, what relationship do you have with these people? What conditions do these people suffer from?

	Relationship with this person (daughter, son, niece, nephew, aunt, uncle, ...) (1)	Which disease? (which allergy, skin condition, asthma, ...) (2)
Person 1 (1)		
Person 2 (4)		
Person 3 (5)		
Person 4 (6)		
Person 5 (7)		
Person 6 (8)		

Q100 Did you use any medication in the past 3 months?

○ Yes (1)

○ No (2)

Q56 If you had to take medication in the past 3 months, please enter this in the text box below. Please list the name, start and stop date and reason for each product.

	Name (1)	Dose (2)	Start date (3)	Stop date (4)	Reason (5)
Medication 1 (3)					
Medication 2 (4)					
Medication 3 (5)					

You have reached the end of this questionnaire. Thanks for completing!

Other relevant notes:

____
____

Questionnaire T12 – Children

Has anything changed in your child's living environment in the past 12 weeks? Eg: change of school, daycare, moving, new hobbies...

- o Yes, describe (shortly) (1) ____
- o No (2)

Personal hygiene:

	Multiple times a day (1)	Daily (2)	> 3x/week (3)	Weekly (4)	Monthly (5)	Seldom (6)	Never (7)
How often does your child shower(1)	211.	212.	213.	214.	215.	216.	217.
How often does your child take a bath (2)	218.	219.	220.	221.	222.	223.	224.
How often does your child use body lotion/milk? (3)	225.	226.	227.	228.	229.	230.	231.
How often does your child use soap when washing (body)? (4)	232.	233.	234.	235.	236.	237.	238.
How often does your child use skin peels or scrubs (5)	239.	240.	241.	242.	243.	244.	245.
How often does your child use antibacterial soap or hand disinfectants (e.g. Dettol, disinfectant hand gel, Stellisept,...? (6)	246.	247.	248.	249.	250.	251.	252.
How often does your child use parfum (8)	253.	254.	255.	256.	257.	258.	259.

Has anything changed in your child's use of care products in the past 12 weeks? E.g. other body lotion, soap, shampoo, ...

- ○ Yes, describe (shortly): (1) ____
- ○ No (2)

How would you describe the general health of your child?

- ○ Very good (1)
- ○ Good (2)
- ○ Moderate (3)
- ○ Bad (4)
- ○ Very bad (5)
- ○ Other: (6) ____

Did your child had to use antibiotics in the past 12 weeks?

- Yes, oral (capsules, tablets,...) (1)
- Yes, local (eg Fucidine in Fucicort of Fucidine Hydrocortisone, Bactrobam,…) (2)
- No (4)

If yes, please give more information on the used antibiotics:

- ◯ Name product (1) ____

- ◯ Start date (2) ____

- ◯ Stop date (3) ____

- ◯ Place(s) one het body where it was used (4)
____

- ◯ Reason treatment (5) ____

Q117 Did your child had to use cortisone in the past 12 weeks?

- ◯ Yes, oral (capsules, tablets,...) (1)

- ◯ Yes, local (eg Pannocort, Cremicort,...) (2)

- ◯ No (4)

If yes, give reason and specifications:

- ◯ Name product (1) ____

- ◯ Start date (2) ____

- ◯ Stop date (3) ____

- ◯ Place(s) one het body where it was used (4)
____

- ◯ Reason treatment (6) ____

Has anything changed in the medication your child is taking in the past 12 weeks?

- ◯ Yes (1)

- ◯ No (2)

If the medication changed, please list the name, start and stop dates and the reason for each product.

	Name (1)	Dose (2)	Start date (3)	Stop date (4)	Reason (5)
Medication 1 (3)					
Medication 2 (4)					
Medication 3 (5)					

Has your child suffered from atopic eczema or other skin conditions in the past 12 weeks?

- Yes (1)
- No (2)

If yes, please provide more information about the skin condition your child suffered from:

- Condition (if possible describe symptoms) (1) ____
- Start date symptoms (2) ____
- Stop date symptoms (if possible) (3) ____
- Treatment (if any) (4) ____

Do you have any other relevant comments for this study?

____

____

You have reached the end of this questionnaire. Thank you very much for completing and participating in this study!

Questionnaire T12 – Adults

Has anything changed in your living environment in the past 12 weeks? Eg: change of work, moving, new hobbies...

- ○ Yes, describe (shortly) (1) ____
- ○ No (2)

Personal hygiene:

	Multiple times a day (1)	Daily (2)	> 3x/week (3)	Weekly (4)	Monthly (5)	Seldom (6)	Never (7)
How often do you shower(1)	260.	261.	262.	263.	264.	265.	266.
How often do you take a bath (2)	267.	268.	269.	270.	271.	272.	273.
How often do you use body lotion/milk? (3)	274.	275.	276.	277.	278.	279.	280.
How often do you use soap when washing (body)? (4)	281.	282.	283.	284.	285.	286.	287.
How often do you use skin peels or scrubs (5)	288.	289.	290.	291.	292.	293.	294.
How often do you use antibacterial soap or hand disinfectants (e.g. Dettol, disinfectant hand gel, Stellisept,...? (6)	295.	296.	297.	298.	299.	300.	301.
How often do you use parfum (8)	302.	303.	304.	305.	306.	307.	308.

Has anything changed in your use of care products in the past 12 weeks? E.g. other body lotion, soap, shampoo, ...

○ Yes, describe (shortly): (1) ____

○ No (2)

How would you describe your general health?

○ Very good (1)

○ Good (2)

○ Moderate (3)

○ Bad (4)

○ Very bad (5)

○ Other: (6) ____

Did you use antibiotics in the past 12 weeks?

- Yes, oral (capsules, tablets,...) (1)
- Yes, local (eg Fucidine in Fucicort of Fucidine Hydrocortisone, Bactrobam,…) (2)
- No (4)

If yes, please give more information on the used antibiotics:

○ Name product (1) ____

○ Start date (2) ____

○ Stop date (3) ____

○ Place(s) on body where used (4)
____

○ Reason treatment (5) ____

Did you use cortisone in the past 12 weeks?

- ◯ Yes, oral (capsules, tablets,...) (1)
- ◯ Yes, local (eg Pannocort, Cremicort,...) (2)
- ◯ No (4)

If yes, give reason and specifications:

- ◯ Name product (1) ____
- ◯ Start date (2) ____
- ◯ Stop date (3) ____
- ◯ Place(s) one het body where it was used (4)
____
- ◯ Reason treatment (6) ____

Did anything change in your medication schedule the last 12 weeks?

- ◯ Yes (1)
- ◯ No (2)

If the medication changed, please list the name, start and stop dates and the reason for each product.

	Name (1)	Dose (2)	Start date (3)	Stop date (4)	Reason (5)
Medication 1 (3)					
Medication 2 (4)					
Medication 3 (5)					

Have you suffered from atopic eczema or other skin conditions in the past 12 weeks?

○ Yes (1)

○ No (2)

If yes, please provide more information about the skin condition your child suffered from:

○ Condition (if possible describe symptoms) (1) ____

○ Start date symptoms (2) ____

○ Stop date symptoms (if possible) (3) ____

○ Treatment (if any) (4) ____

Do you have any other relevant comments for this study?

____

____
